# A Practical Method for Synthesizing Iptacopan

**DOI:** 10.3390/molecules29102289

**Published:** 2024-05-13

**Authors:** Zhiwei Tang, Shaojie Chu, Xuesong Wu, Shaoxin Chen, Likuo Chen, Jiawei Tang, Hongbo Wang

**Affiliations:** National Key Laboratory of Lead Druggability Research, Shanghai Institute of Pharmaceutical Industry, China State Institute of Pharmaceutical Industry, 285 Gebaini Road, Pudong, Shanghai 201203, China; aomori_vale@163.com (Z.T.); 17872135592@163.com (S.C.); wuxuesong@sinopharm.com (X.W.); sxzlb@263.net (S.C.); 13375337692@163.com (L.C.)

**Keywords:** Iptacopan, ketone reductase, complement pathway

## Abstract

Iptacopan, the first orally available small-molecule complement factor B inhibitor, was developed by Novartis AG of Switzerland. Iptacopan for the treatment of PNH was just approved by the FDA in December 2023. Other indications for treatment are still in phase III clinical trials. Iptacopan is a small-molecule inhibitor targeting complement factor B, showing positive therapeutic effects in the treatment of PNH, C3 glomerulonephritis, and other diseases. Although Iptacopan is already on the market, there has been no detailed synthesis process or specific parameter report on the intermediates during the synthesis of its compounds except for the original research patent. In this study, a practical synthesis route for Iptacopan was obtained through incremental improvement while a biosynthesis method for ketoreductase was used for the synthesis of the pivotal intermediate **12**. Moreover, by screening the existing enzyme library of our research group on the basis of random as well as site-directed mutagenesis methods, an enzyme (M8) proven to be of high optical purity with a high yield for biocatalectic reduction was obtained. This enzyme was used to prepare the compound benzyl (2*S*,4*S*)-4-hydroxy-2-(4-(methoxycarbonyl)-phenyl)-piperidine-1-carboxylate) white powder (36.8 g HPLC purity: 98%, *ee* value: 99%). In the synthesis of intermediate **15**, the reaction was improved from two-step to one-step, which indicated that the risk of chiral allosterism was reduced while the scale was expanded. Finally, Iptacopan was synthesized in a seven-step reaction with a total yield of 29%. Since three chiral intermediate impurities were synthesized directionally, this paper lays a solid foundation for the future of pharmaceutical manufacturing.

## 1. Introduction

As a precursor of C3 activator, complement factor B is integral in the complement bypass activation pathway, being mainly synthesized by liver and macrophage cells [1]. It is an important component involved in complement bypass activation as well as body defense, which plays a vital role in tissue and cell damage and inflammation. Iptacopan, the first targeted small-molecule inhibitor of complement factor B for the treatment of PNH, has been approved by the FDA. Other indications are used in Phase III clinical trials for the treatment of C3 glomerulonephritis and lgA nephropathy [2]. In Phase II clinical trials, Iptacopan was proven to be effective, with the advantages of high safety, good tolerability, and high oral bioavailability. Therefore, an ever-increasing number of people have begun to take note of the need for a concise and efficient approach to synthesize Iptacopan.

**Scheme 1:**
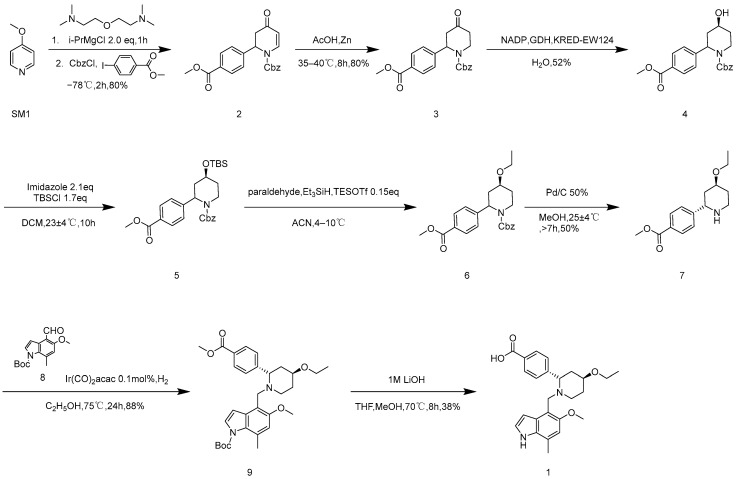
Original synthetic route 1.

**Scheme 2:**
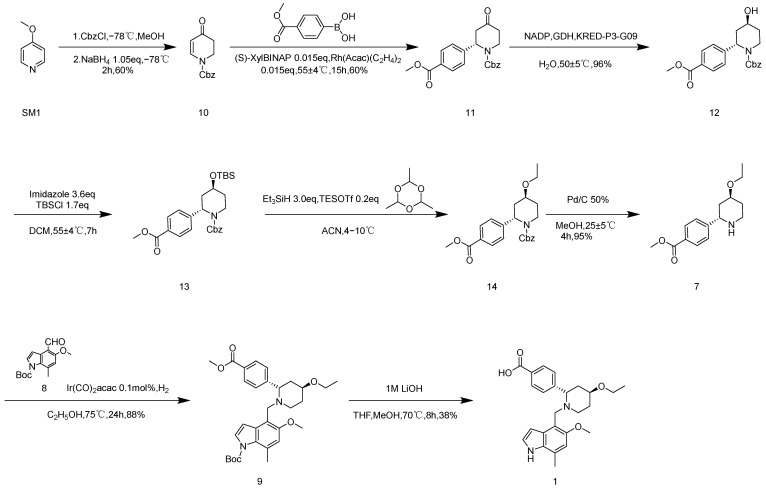
Original synthetic route 2.

It can be seen in Scheme 1 that the original research route consists of 14 steps [3,4]. Both the synthesis routes in Scheme 1 and Scheme 2 have some shortcomings that make them unsuitable for industrial production. The total yield of route 1 is 7.3%, there are lengthy steps in this route, and the chirality need to be split to obtain the monomer. The total yield of route 2 is 16.3% and has high optical purity, but the route is long, and the conditions of some steps are not suitable for industrial production. For route 1, Kumada coupling is used for **SM 1** to synthesize **2**, which is reduced by zinc powder/acetic acid to obtain compound **3**, which is catalyzed by an enzyme to collect compound **4**; then, ethylated product **6** is obtained via a two-step reaction. Since **6** is benzyl chloroformate stripped by palladium carbocatalytic hydrogenation, product **7** is obtained by the chiral resolution. Moreover, **7** and **8** are reduced and aminated with iridium catalyst groups to obtain **9**. Finally, under the condition of 1 M lithium hydroxide, the methyl ester group is hydrolyzed and the Boc group is stripped to obtain **1**. As shown in Scheme 2, **10** is prepared by salting benzyl chloroformate and reducing with sodium borohydride, then **11** is produced by rhodium catalyzed coupling. Thereafter, enzymatic catalysis is used to obtain compound **12**; ethylated product **14** is obtained through a two-step reaction, and the protective group of benzyl chloroformate is removed by palladium carbocatalytic hydrogenation to obtain **7**. Then, **7** and **8** are reduced and aminated using iridium catalyst groups to obtain **9**. In conclusion, under the condition of 1 M lithium hydroxide, the methyl ester group is hydrolyzed and the Boc group is stripped to obtain **1**. Compared to route 1, in route 2, Iptacopan is obtained quickly, efficiently, with reduced energy consumption, and at low cost. In this study, the temperature of the ultra-low temperature reaction is studied. The conditions for metal-catalyzed reactions are improved, and enzyme-catalyzed reaction conditions are optimized. At the same time, we attempt to stack reaction steps and improve the reaction yield.

## 2. Results and Discussion

### 2.1. Optimization of Reaction Conditions for Benzyl 4-Oxo-3,4-dihydropyridine-1(2H)-carboxylate

**Scheme 3:**
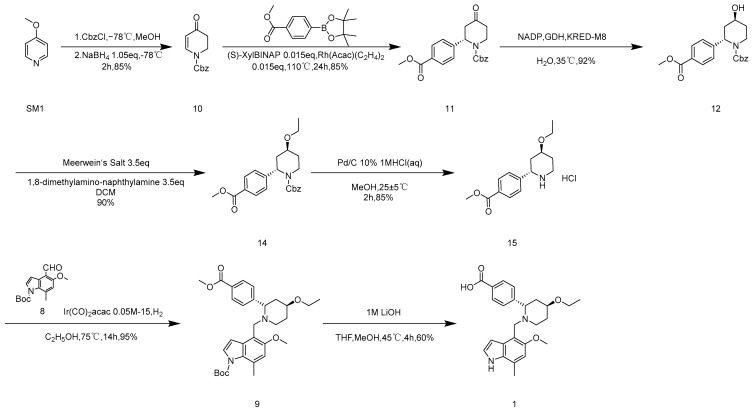
Optimized route to Iptacopan hydrochloride.

As explained in Scheme 3, using commercially available 4-methoxypyridine (**SM1**) as the starting material, the original synthesis conditions were tested while the target yield was not reached. The mechanism study pinpointed that methanol and benzyl chloroformate would produce an esterification reaction [5], which would affect the actual dosage of benzyl chloroformate. In this paper, the mixed solvent of tetrahydrofuran and methanol was first replaced with tetrahydrofuran as a solvent, and the yield was increased to 62.4% (as shown in Table 1, entry 4). Because temperature plays a crucial role in the initiation of sodium borohydride and it has been found in experiments that elevated temperature may produce over-reduction products that reduce ketone carbonyl groups to hydroxyl groups, reaction temperature is also an important factor. In this paper, the overreduction products were separated and their structures were confirmed, and their structures were determined to be impurities with both ketone carbonyl and olefin double bonds being reduced (Figure S1. MS data of 22). After **10** was reacted with sodium borohydride at room temperature, 0 °C, and −20 °C, respectively, it was found that **10** was stable at −20 °C. Therefore, this study tried to use −20 °C as the reaction condition to prepare **10**. Under a mild reaction condition of −20 °C (as shown in Table 1 and entry 7), the reaction yield of **10** rose to 84.5%, which finally achieved 100 g scale amplification.

### 2.2. Effect of Reaction Conditions on the Synthesis of Compound **11**

The conditions were explored owing to the barely satisfied results of the synthesis of compound **11** by the original patented method. The stability of the metal catalyst was analyzed, suggesting the catalyst was sensitive to oxygen and protons [6,7,8]. In view of the existence of a boric acid group, alkali was added to the system to react with an acid-binding agent. At the same time, we screened the type of base. We found that organic bases could not facilitate the reaction and potassium carbonate increased the hydrolysis of methyl ester groups. Therefore, it was determined that cesium carbonate was conducive to the proceeding of the reaction. The reaction could be carried out normally, whereas the yield was low. Considering the price and stability of rhodium catalyst [9,10,11], attempts to replace palladium catalyst did not obtain good results [12,13,14]. To this end, the raw material **16** was substituted by **17**, and the free proton was solved from the root with the yield improvement. The reaction conditions were obtained using substrate **17** (2.5 eq), reaction solvent (1, 4-dioxane: H_2_O = 5:1), and cesium carbonate (3 eq), as shown in Table 2. Since 1,4-dioxane is a class 2B carcinogen, replacing the reaction solvent should be considered. Solvents with comparable polarity and chemical properties to 1,4-dioxane, such as tetrahydrofuran, acetonitrile, and toluene with good substrate solubility, were selected to react under the same conditions, and the results showed that toluene as a solvent was almost equivalent to 1,4-dioxane as a solvent. The reaction conditions were obtained using substrate **17** (2.5 eq), reaction solvent (Tol: H_2_O = 5:1), and cesium carbonate (3 eq), as shown in Table 2. The principal impurities of the reaction were identified and analyzed, and it was found that the ester group of **11** underwent a hydrolysis reaction and was the main by-product when heated and refluxed under alkaline conditions (Figure S2. MS data of 23), and it could be esterified to obtain compound **11** after being recovered during the purification process. Under this condition, 10 g amplification is achieved.

### 2.3. Screening of Ketoreductases

**Figure 1:**
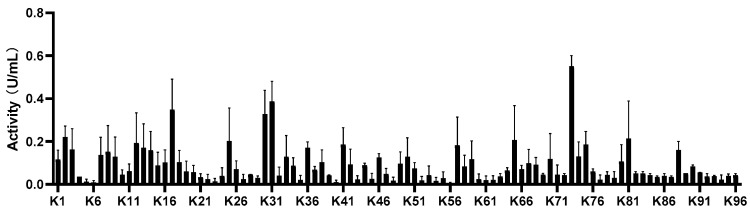
Activity screening of 96 enzymes’ reaction conditions: Activity screenings were performed by adding 190 μL of enzymatic assay solution (containing the 0.2 mM substrate **11** in 10% DMSO, 1 mM NAD(P)H in 100 mM, pH 7.0 phosphate buffer).

The cis-inverse ratio of enzymatically induced compound **12** reported in the literature is 90:10, which had an effect on the chirality of the terminal product. Therefore, the synthesis of **12** induced a second chiral center, which was one of the major steps in this route. However, the optical purity was low after attempting to induce chirality by employing chemical approaches. In this paper, 96 ketoreductases (K1–K96, as shown in Figure 1) presented in our research group were screened, while **7** ketoreductases suitable for **11** were obtained with their *ee* values measured. Among them, enzymes K2, K17, K30, K31, and K73 had high reaction activity. Accordingly, a single isomer was reduced by enzyme K73, as stated in Table 3, and another single isomer was reduced by enzyme K2. The product reduced by enzyme K73 was a wild-type ketoreductase from *Exiguobacterium* sp. F42 named WTEA. WTEA could asymmetrically reduce various bulky *Exiguobacterium* sp. ketones [12].

### 2.4. Determination of the Absolute Configuration

To determine the absolute configuration of the molecule, the **15** single crystals were prepared by slow volatilization. Subsequently, it was confirmed that the single crystals belonged to the orthorhombic crystal system. The crystal size was 0.20 × 0.06 × 0.04 mm, and the crystal density was 1.247 mg/m^3^. The absolute structure of compound 15 can be determined from the Flack’s parameter: 0.045. The SXRD results suggest that compound 15 is crystallized in the orthorhombic space group P2_1_2_1_2_1_ and possesses four formula units per unit cell (Z = 4). For the single crystal of compound **15**, an asymmetric unit containing **7** and HCl with a ratio of 1:1 emerged. Table S2 summarizes the crystal parameters, data collection, and refinement details of the salt.

**Figure 2:**
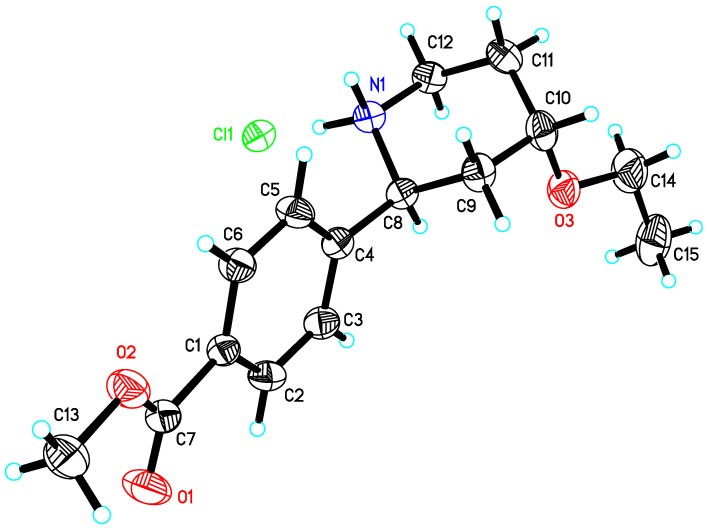
Single-crystal XRD structure of **15**.

In this paper, the enantiomers of **11** were prepared using R-type chiral ligands and separated in the liquid phase to determine the *ee* value of **11** (Figure S8). Compound 11 was used to prepare Rac-12 through sodium borohydride reduction; this was separated in the liquid phase, and the *ee* value of **12** was determined (Figure S9). It was prepared by a two-step reaction to obtain **15**, and the absolute configuration of **15** was obtained through a single-crystal culture (as shown in Figure 2). Meanwhile, the chiral configuration reduced by this enzyme was determined as the required configuration.

### 2.5. Screening of a WTEA Variant Library

**Figure 3:**
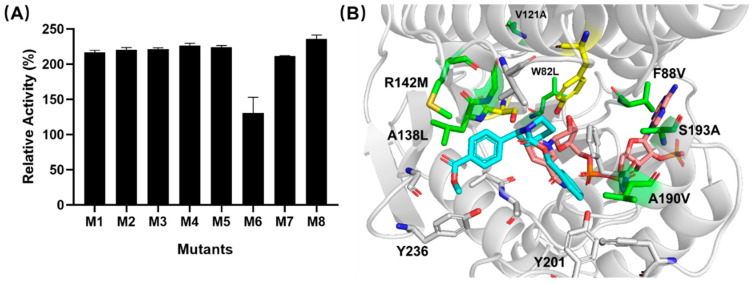
(**A**) Results of screening the mutation library of WTEA reaction conditions: Activity screenings were performed by adding 190 μL of enzymatic assay solution (containing the 0.2 mM substrate **11** in 10% DMSO and 1 mM NAD(P)H in100 mM in pH 7.0 phosphate buffer). (**B**) Substrate-binding pocket of M8.

In previous studies, we constructed a high-quality mutation library through crystal structural analysis, CSR-SALAD, HotSpot Wizard prediction, and deep multiple-sequence alignment analysis [15]. In order to find more active WTEA mutants, **11** was reacted with the iterative combination mutation library of WTEA. This library [15] was constructed by introducing mutations at multiple site residues (W82, F88, V121, A138, R142, A190, S193, Y201, N204, K206, and K207) in order to alter the size and shape of the substrate binding pocket. The full list of the variants as well as the screening results are given in Table S1 and Figure 3A (see Supplementary Information for additional details). Variant M8 (W82L/F88V/V121A/A138L/R142M/A190V/S193A) reflected the highest activity, namely, a 2.4-fold enhancement compared to the wild-type enzyme. To investigate the interaction between enzymes and substrates, we simulated the protein structure of M8 by applying the webtool SWISS-MODEL with the crystal structure of ketoreductase (PDB entry 7E3X) from *Exiguobacterium* as a template. Given the high level of sequence identity (98%) within the M8 and 7E3X, the model obtained from homology modeling was used to dock with substrate **11** through AutoDock. In this simulated activity pocket (Figure 3B), it was obvious that substrate **11** was docked into the region near the NADPH. Expectedly, the binding affinity between the substrate and enzyme was promoted by the mutations A138L, R142M, A190V, and S193A. The catalytic activity improvement of mutation F88V might have enhanced the affinity of the cofactor NADPH. The two distal mutations (W82L/V121A) more than 10 Å away from the active site might affect enzyme activity by enhancing loop flexibility near catalytic pocket D [15,16]. Therefore, we selected mutant M8 for the follow-up study.

### 2.6. Optimization of the Reaction Conditions of Enzyme Catalysis: Optimization of the Substrate Concentration

**Figure 4:**
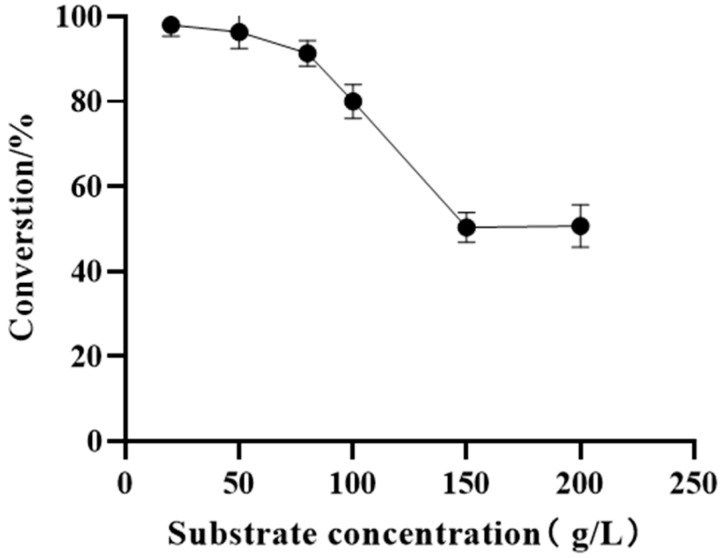
Effect of substrate concentration on reduction **11**. Reaction conditions: various concentrations of substrate **11** (including 20, 50, 80, 100, 150, and 200 g/L), 15 g/L frozen cell pellets of M8, 7.5 g/L frozen cell pellets of GDH, 2.0 equiv. glucose and PBS buffer (100 mM, pH 7.0) in a 2 mL reaction system for 18 h at 25 °C and 220 rpm.

Optimization of the reaction parameters not only improved the conversion efficiency but also minimized production costs to meet industrial requirements. As a contributing factor, the substrate concentration should be considered in industrial production. The substrate loading study was carried out in the concentration range of 20−200 g/L to investigate the catalytic capacity. When the substrate concentration was observed to be below 80 g/L, the compound conversion rate remained above 90%. However, there was a sharp decline in the conversion rate as the substrate concentration was increased to 150 g/L. Our previous research determined that the pH and temperature are essential for the enzyme activity improvement. Thus, the process optimization for the reaction conditions, including the pH, temperature, and catalyst loading, was subsequently performed (Figure 4).

### 2.7. Selection and Optimization of the Reaction Co-Solvent

**Figure 5:**
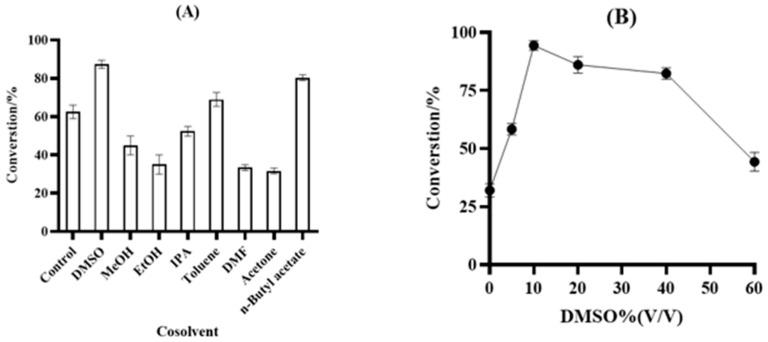
(**A**) Comparison of the performance of different cosolvents. Reaction condition: 100 g/L ketone **11**, 15 g/L frozen cell pellets of M8, 7.5 g/L frozen cell pellets of GDH, 2.0 equiv. of glucose, and PBS buffer (100 mM, pH 7.0) in a 2 mL reaction system for 18 h at 25 °C and 220 rpm in 20% solvent. (**B**) Effect of the different concentrations of DMSO on the bioreduction system. Reaction condition: 100 g/L ketone **11**, 15 g/L frozen cell pellets of M8, 7.5 g/L frozen cell pellets of GDH, 2.0 equiv. of glucose, and PBS buffer (100 mM, pH 7.0) in a 2 mL reaction system for 18 h at 25 °C and 220 rpm in 0% DMSO (*v*/*v*) to 60% DMSO.

The type and amount of solvent had a considerable impact on the activity of the enzyme; therefore, the types and dosage of solvents were studied in this paper. Under the conditions of 100 g/L ketone **11**, 15 g/L frozen cell pellets of M8, 7.5 g/L frozen cell pellets of GDH, 2.0 equiv. of glucose, and PBS buffer (100 mM, pH 7.0) in a 2 mL reaction system for 18 h at 25 °C and 220 rpm in 20% solvent, different solvent types such as DMSO, MeOH, EtOH, IPA, toluene, DMF, acetone, and n-butyl acetate were selected for screening, as shown in Figure 5A. Thereafter, it was observed that solvents such as methanol, ethanol, and DMF were not conducive to the occurrence of enzymatic reactions. DMSO and n-butyl acetate exercised slight influence on the enzymatic reaction, and, thus, DMSO with less influence and a higher conversion rate was selected to optimize the reaction in this paper. Under the same reaction conditions illustrated above, the DMSO volume ratio was gradually increased from 0% to 60%, and the concentration was determined based on the conversion data in Figure 5B. Finally, 10% DMSO was determined to be the most appropriate amount of reaction solvent.

### 2.8. Optimization of the pH and Temperature

**Figure 6:**
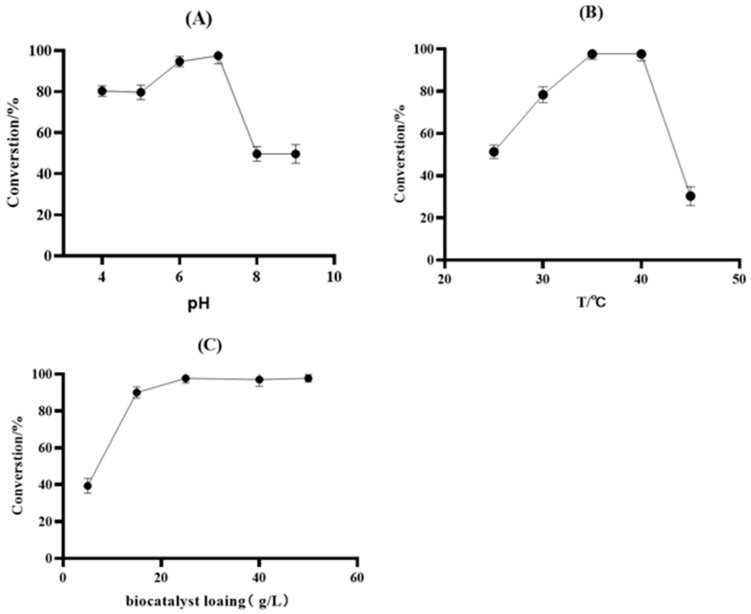
(**A**) Effect of the pH on the bioreduction system. Reaction conditions: the reactions were performed with 20 g/L compound **11**, 5 g/L frozen cell pellets, 4 g/L GDH frozen cell pellets, and 2.0 equiv. of glucose in a 2 mL reaction system for 30 min at 25 °C and 220 rpm in different pH buffer solutions, including 100 mM sodium citrate (pH 4 and 5), 100 mM potassium phosphate (pH 6, 7, and 8), and 100 mM Tris−HCl buffers (pH 9). (**B**) Effect of the temperature on the bioreduction system. Reaction conditions: the temperature study was carried out with 20 g/L ketone 2, 5 g/L frozen cell pellets, 4 g/L GDH frozen cell pellets, 2.0 equiv. of glucose, and PBS buffer (100 mM, pH 7.0) in a 10 mL reaction system for 30 min at 220 rpm. (**C**) Effects of the biocatalyst loading on conversion by frozen cell pellets. Reaction conditions: various KRED-M8 frozen cell pellets in the range of 5–50 g/L, 25 g/L compound **11**, 20 g/L GDH frozen cell pellets, and PBS buffer (100 mM, pH 7.0) in a 10 mL reaction system at 35 °C and 220 rpm.

Both the pH and temperature had an impact on the enzymatic activity. The pH tolerance study regarding the reaction system including KRED-M8 and GDH was conducted to adjust the pH to 4–9, as shown in Figure 6A. The results revealed that when the pH was below 5, the catalytic activity of the enzymes somewhat decreased, while above pH 7, the enzyme activity decreased drastically. The optimum pH was 6–7. Figure 6B indicates that the optimum temperature for the reaction system was determined to be 35–40 °C. It could be seen that the conversion plunged at temperatures above 40 °C. Therefore the optimized temperature chosen was 35 °C.

### 2.9. Optimization of the Biocatalyst Loading

Since the conversion was merely 65−70% under the existing conditions, we increased the KRED-M8 frozen cell pellet dosage from 5 to 50 g/L. As shown in Figure 6C, the reaction can proceed completely with 25 g/L frozen cell pellets, and for that reason, 25 g/L frozen cell pellets were chosen. Considering that the high conversion rate and 99.9% *ee* of M8 met the synthesis requirements of compound **12**, further research was conducted on the catalytic process of M8.

### 2.10. Scaling up the Enzymatic Process

This biotransformation process was scaled up to 40 g. At the beginning of the reaction, 25 g frozen cell pellets were charged to start the reaction. The reaction conversion reached 100% after 12 h and increased slowly. Then, product **12** was isolated, followed by crystallization with a 92% yield with 99.9% HPLC purity and 99.5% *ee*. In terms of the comparison between the results of chemical-induced chirality and enzyme-induced chirality (Table 4), we envisoned that our enzymatic process had the potential to be a cost-effective and environmentally friendly process suitable for industrial applications.

### 2.11. Optimization of the Synthesis of Compound ***14***

In line with the original research route, the yield of the synthesis of **13** was theoretically quantified. However, the conversion and yield of compound **14** did not appear promising, as described in Table 5, entry 5. Hence, N-ethylpyridinium tetrafluoroborate, trifluoromethanesulfonic anhydride/EtOH, and sodium were changed to shorten the synthesis route; hydrogen/iodoethane and triethyloxonium tetrafluoroborate **12** were used to directly prepare **14** (entry 1–4). Among them, only triethyloxonium tetrafluoroborate had an orate result, raising the conversion rate to 99.9% and the yield to 74.6%. After confirming the use of Meerwein’s salt and a proton sponge in combination [17,18], the necessity of the proton sponge was also verified in this paper, and it was found that the reaction conversion rate was 48.5% when the proton sponge was not added (entry 6). The results were consistent with the use of other organic bases to replace the proton sponge, with the same results when the proton sponge was not added (entry 7). The proton sponge was determined to be a reagent to facilitate the reaction, and Meerwein’s salt and the proton sponge were finally selected as ethylation reagents. This step shortened the reaction step and improved the conversion and yield of the reaction, and thus the production was scaled up to 10 g.

## 3. Conclusions

In this paper, a practical and efficient synthesis method for Iptacopan was developed, which applied 4-methoxypyridine as a competitive starting material. Moreover, energy consumption was reduced by changing the ultra-low temperature reaction conditions of the first step, while the stability of the metal catalyst was enhanced by adding alkali and using borate as the reaction reagent. Hence, **11** with high optical purity was obtained. In the construction of the secondary chiral center, we employed biological methods in an effort to reduce ketones with modified enzyme M8. Additionally, the temperature, pH, and dosage of the enzymatic reaction were preliminarily studied. After screening, the best reaction conditions were determined to be under 35 °C, controlled between pH 6 and 7, using 10% DMSO as a co-solvent, and using 25 g/L of frozen stem cell M8 to catalyze 80 g/L of substrate for the reaction. One-step synthesis was used to synthesize **14**, which reduced the risk of chiral allostery and shortened the synthesis step. Finally, the reaction step was shortened, a seven-step reaction was used, and a new route with a total yield of 29.0% was obtained, which is suitable for industrial production. Importantly, our study, provided novel insights into the industrialization of Iptacopan at later stages, presented the efficient total synthesis of Iptacopan with high optical purity, located the isomer impurities of the intermediates, and built a synthesis route more suitable for industrial production.

## 4. Experimental Section

### 4.1. Materials and Methods

Glucose was purchased from Aladdin (Shanghai, China) while the other chemicals were gained commercially at an analytical grade. The ^1^H NMR and ^13^C NMR spectra were recorded using a Bruker AVANCE III 400 MHz spectrometer employing DMSO-d6 and CDCl_3_ as solvents. The ^1^H NMR chemical shift data and the ^13^C NMR chemical shift data were reported for δ (ppm) relative to tetramethylsilane as the internal standard and δ (ppm) relative to DMSO-d6 and CDCl_3_, respectively. The reaction was monitored by HPLC (Waters 2998–2695), and the *ee* was determined by chiral HPLC (Waters 2998–2695). The ketone reductases of K1–K96 were all constructed by the research group in the early stage [18].

### 4.2. Screening of the Variant(s) Library

Recombinant *E. coli* colonies were placed into 400 μL of LB liquid medium (50 μg/mL kanamycin final concentration) and cultivated overnight at 37 °C and 1000 rpm. Then, 20 μL of preculture was inoculated into 600 μL of LB liquid medium and incubated at 37 °C and 1000 rpm until the OD_600_ reached 0.6. Subsequently, the culture was induced with isopropyl β-D-1-thiogalactopyranoside (1 mM final concentration) and continued overnight at 28 °C and 1000 rpm. The cells were harvested by centrifugation for 20 min at 2000× *g*. After the supernatant was discarded, 400 μL phosphate buffer (100 mM, pH 7.0) with the addition of 1000 U of lysozyme was added to each well to resuspend the cells and shaken at 30 °C for 30 min. The crude lysate was centrifuged (3000× *g*, 30 min) at 4 °C. The 10 μL supernatant was transferred to a 96-well plate and used for the high-throughput assay. Activity screenings were performed by adding 190 μL of enzymatic assay solution (containing the 0.2 mM substrate **11** in 10% DMSO and 1 mM NADPH in 100 mM of pH 7.0 phosphate buffer). The activity of each mutant was measured by the decrease in absorbance of NADPH at 340 nm.

### 4.3. Culture of E. coli/pET-28a (+)-M8 Cells in a 10 L Bioreactor

*E. coli*/pET-28a (+)-M8 cells were precultured overnight in 150 mL of LB medium containing 50 μg/mL kanamycin at 37 °C. Then, the seed culture was transferred to a 10 L bioreactor containing 7 L of fermentation medium (12 g/L yeast extract, 12 g/L soybean peptone, 3 g/L NaCl, 10 g/L glycerol, 2 g/L K_2_HPO_4_, 0.5 g/L MgSO_4_·7H_2_O, 50 μg/mL kanamycin, and pH 7.0). As the optical density of the culture measured at a wavelength of 600 nm reached 10.0, the cultures were cooled to 25 °C, and 168 mg of IPTG was added to induce the expression for 20 h. Stored at −20 °C, the recombinant *E. coli*/pET-28a (+)-M8 cells were harvested by centrifugation (4000× *g*, 30 min, 4 °C). Likewise, the *E. coli*/pET-28a (+)-M8-GDH cells were prepared by this method.

### 4.4. Synthesis of Benzyl 4-Oxo-3,4-dihydropyridine-1(2H)-carboxylate

**SM1** (10 g, 92 mmol) was placed in a 100 mL four-mouth bottle with three nitrogen displacements. Since the internal temperature was lowered to −20 °C, 10 mL of methanol was added to the reaction bottle to mix well. Keeping the temperature below −20 °C, an anhydrous tetrahydrofuran (10 mL) solution of benzyl chloroformate (16 g, 100 mmol) was slowly dropped to the reaction bottle. Next, the reaction liquid turned into a white turbid liquid for 1 h, and sodium borohydride solid was added in batches. On the strength of the rising temperature, the reaction liquid turned into yellow turbid liquid and was then monitored by TLC (PE: EA = 3:1 R_f_ = 0.4). Upon completion, 3 wt.% HCl(aq) was added at a low temperature to quench the reaction with a significant warming phenomenon. DCM was added for extraction and then stratified; then, the organic phase was washed twice with 3 wt.% Na_2_CO_3_(aq) and then washed once with saturated sodium chloride. Finally, the organic phase was dried with anhydric sodium sulfate. The light-yellow oil was concentrated under reduced pressure while 18 g of the white solid was obtained by adding methyl tert-butyl ether for crystallization at a lower temperature. Yield 85%, mp. 65.3–66.7 °C; HRMS(ESI) *m*/*z*: calculated for C_13_H_13_NO_3_^+^ [M + H]^+^ 232.09692, [M + Na]^+^ 254.07874, found 232.09692, 254.07874. ^1^H NMR (400 MHz, CDCl3) δ 7.75 (s, 1H), 7.32–7.26 (m, 5H), 5.24 (d, *J* = 6.2 Hz, 1H), 5.17 (s, 2H),3.97–3.90 (m, 2H), 2.49–2.41 (m, 2H). ^13^C NMR (101 MHz, CDCl_3_) δ 207.2 (s), 193.3 (s), 134.9 (s), 107.8 (s), 69.1 (s), 67.6 (s), 43.1 (s), 42.6 (s), 41.1 (s), 35.7 (s).

### 4.5. Synthesis of Benzyl (S)-2-(4-(Methoxycarbonyl)-phenyl)-4-oxopiperidine-1-carboxylate

In a 1 L three-mouth bottle containing compound **10** (10 g, 43.28 mmol), Pinalol 4-methoxycarbonylphenylborate (45.38 g, 173.12 mmol), (*S*)-2,2′-bis[bis(3,5-dimethylphenylphosphine)]-1,1′ -binaphthalene ((*S*)-XylBINAP) (2.39 g, 3.25 mmol), Bis(η2-ethene)(2,4-pentanedionato-κO,κO′)rhodium (Rh(Acac)(C_2_H_4_)_2_)(336.0 mg, 1.30 mmol), and cesium carbonate (42.27 g, 129.73 mmol), Tol (200 mL) and water (40 mL) were added while nitrogen was substituted three times; then, the reaction liquid was heated to the reflux state internal temperature of 92 °C. Consequently, the reaction liquid became dark wine-red. TLC monitoring (PE: EA = 3:1 R_f_ = 0.3) suggested that the reaction was basically completed for 48 h. After the completion of the reaction, the reactant was restored to room temperature and concentrated under reduced pressure. After being added to dissolve, the ethyl acetate was pumped and filtered; the filter cake should be washed until white. Using column chromatography, the filtrate was concentrated under reduced pressure and then recrystallized with n-heptane: methyl tert-butyl ether = 3:1 to obtain 8.1 g of white solid and 5.2 g of yellow oil; yield 85%, mp. 81.2–82.5 °C; HRMS(ESI) *m*/*z*: calculated for C_21_H_21_NO_5_^+^ [M + H]^+^ 368.14929, [M + Na]^+^ 390.13097, found 368.14929, 390.13097.^1^H NMR (600 MHz, CDCl3) δ 7.92 (d, *J* = 8.3 Hz, 2H), 7.33–7.20 (m, 7H), 5.75 (s, 1H), 5.21–5.08 (m, 2H), 4.24 (s, 1H), 3.84 (s, 3H), 3.16 (s, 1H), 2.95–2.79 (m, 2H), 2.53–2.43 (m, 1H), 2.32 (d, *J* = 16.1 Hz, 1H). ^13^C NMR (101 MHz, CDCl_3_) δ 206.7 (s), 166.6 (s), 155.4 (s), 136.0 (s), 130.2 (s), 129.7 (s), 128.6 (s), 128.4 (s), 128.1 (s), 126.6 (s), 68.0 (s), 54.7 (s), 52.2 (s), 44.2 (s), 40.5 (s), 39.2 (s), 24.9 (s).

### 4.6. Synthesis of Benzyl (2S,4S)-4-Hydroxy-2-(4-(methoxycarbonyl)-phenyl)-piperidine-1-carboxylate

Compound **11** (40 g, 217.76 mmol) was dissolved in DMSO (200 mL), 0.1 M phosphate buffer (1800 mL, pH = 7.0), NADP^+^ (0.8 g), and KRED-M8 frozen cell pellets (50 g), and GDH frozen cell pellets (40 g) were added successively. The reaction was performed in a constant-temperature shaker at 220 rpm and 35 °C. The reaction was monitored by TLC (PE:EA = 1:1 R_f_ = 0.3) until it was basically completed in 24 h. Dichloromethane (1000 mL) was added to quench the reaction, from which the product was extracted and then centrifuged. The organic phase was concentrated under reduced pressure and was substituted with ethyl acetate solvent. After being washed with saturated salt water three times, the solvent was dried with anhydrous sodium sulfate, filtered, and concentrated into a white solid weighing 36.8 g; yield 92%, mp. 90.5–92.0 °C; HRMS(ESI) *m*/*z*: calculated for C_21_H_23_NO_5_^+^ [M + H]^+^ 370.16514, [M + Na]^+^ 392.14642, found 370.16514, 392.14642. ^1^H NMR (600 MHz, CDCl3) δ 7.92 (dd, *J* = 8.3, 4.2 Hz, 2H), 7.27–7.16 (m, 7H), 5.64–5.31 (m, 1H),5.14–5.04 (m, 2H), 4.11 (s, 1H), 3.84 (d, *J* = 5.9 Hz, 3H), 3.34 (td, *J* = 13.3, 3.3 Hz, 1H), 2.76 (d, *J* = 2.6 Hz, 1H), 2.35 (d*, J* = 14.6 Hz, 1H), 1.80 (ddd*, J* = 13.4, 8.2, 3.1 Hz, 2H), 1.61 (d, *J* = 12.6 Hz, 1H). ^13^C NMR (101 MHz, CDCl_3_) δ 166.8 (s), 155.9 (s), 144.7 (s), 136.5 (s), 130.1 (s), 128.9 (s), 128.6 (s), 128.2 (s), 127.9 (s), 126.2 (s), 67.6 (s), 64.9 (s), 53.8 (s), 52.2 (s), 39.3 (s), 37.1 (s), 34.9 (s), 29.7 (s).

### 4.7. Synthesis of Benzyl (2S,4S)-4-Ethoxy-2-(4-(methoxycarbonyl)-phenyl)-piperidine-1-carboxylate

Compound **12** (10 g, 27 mmol) was placed in a 50 mL three-mouth bottle and dissolved with dichloromethane. After being completely dissolved, 1,8-dimethylamino-naphthylamine (20.3 g, 94.5 mmol) was added to the reaction solution, which turned brown and yellow. The triethyloxonium tetrafluoroborate solid (18.0 g, 94.5 mmol) was quickly weighed and added to the reaction bottle under a nitrogen drum to produce a large amount of solid after 5 min. Completion of the reaction was monitored by TLC (first PE:EA = 3:1, then PE:EA = 1:1 R_f_ = 0.6). After the reaction was completed, the filter cake was pumped and washed with ethyl acetate until it was white, then the filtrate was washed once with 1 M HCl(aq) and then washed with saturated salt water. Finally, the organic phase was condensed under pressure to obtain 9.6 g of colorless oil; crude yield 90%; HRMS(ESI) *m*/*z*: calculated for C_23_H_27_NO_5_^+^ [M + H]^+^ 398.19637, [M + Na]^+^ 420.17815, found 398.19637, 420.17815. ^1^H NMR (400 MHz, CDCl3) δ 8.00 (d, *J* = 8.4 Hz, 2H), 7.32 (dt, *J* = 17.7, 7.9 Hz, 7H), 5.68 (s, 1H), 5.19 (s, 2H), 4.27 (d, *J* = 13.4 Hz, 1H), 3.92 (s, 3H), 3.56–3.42 (m, 2H), 3.40–3.28 (m, 1H), 2.84 (td, *J* = 13.6, 2.9 Hz, 1H), 2.65 (d, *J* = 13.4 Hz, 1H), 2.06–1.91 (m, 1H), 1.84 (ddd, *J* = 13.4, 11.4, 5.8 Hz, 1H), 1.54–1.40 (m, 1H), 1.18 (t, *J* = 7.0 Hz, 3H). ^13^C NMR (101 MHz, CDCl_3_) δ 166.8 (s), 155.9 (s), 145.0 (s), 136.5 (s), 130.1 (s), 128.9 (s), 128.5 (s), 128.1 (s), 127.9 (s), 126.1 (s), 71.7 (s), 67.6 (s), 63.3 (s), 53.8 (s), 52.1 (s), 39.4 (s), 34.5 (s), 31.8 (s), 15.6 (s).

### 4.8. Synthesis of Methyl 4-((2S,4S)-4-Ethoxypiperidin-2-yl)-benzoate Hydrochloride

Compound **14** (14.5 g, 36.5 mmol) was dissolved in a 100 mL single-mouth bottle with methanol (40 mL) and 1 M HCl (22 mL, 43.8 mmol). Then, 10% Pd/C (1.45 g, 10%M-14) was added to take the place of nitrogen and hydrogen one time and three times, respectively. The reaction was monitored by TLC (DCM:MeOH = 10:1 R_f_ = 0.3) after 12–15 h. Subsequently, the filtrate was pumped and concentrated under reduced pressure to obtain a white solid. A white solid powder weighing 9.3 g was then obtained using methyl tert-butyl ether slurry to remove impurities. Yield 85%; mp. 205.5–207 °C; HRMS(ESI) *m*/*z*: calculated for C_15_H_21_NO_3_^+^ [M + H]^+^ 264.15926, found 264.15926. ^1^H NMR (600 MHz, CDCl3) δ 9.74 (s, 2H), 8.00 (d, *J* = 7.6 Hz, 2H), 7.69 (d, *J* = 7.8 Hz, 2H), 4.41 (s, 1H), 3.92 (s, 3H), 3.80 (s, 1H), 3.52–3.40 (m, 2H), 3.16 (s, 1H), 2.90 (s, 1H), 2.28–2.06 (m, 3H), 1.92(d, *J* = 14.1 Hz, 1H), 1.21 (t, *J* = 6.9 Hz, 3H). ^13^C NMR (101 MHz, MeOD) δ 166.3 (s), 141.3 (s), 131.0 (s), 130.0 (s), 127.4 (s), 69.1 (s), 63.7 (s), 54.9 (s), 51.5 (s), 40.5 (s), 33.6 (s), 25.5 (s), 14.5 (s).

### 4.9. Synthesis of Tert-Butyl 4-(((2S,4S)-4-ethoxy-2-(4-(methoxycarbonyl) phenyl) piperidin-1-yl)-methyl)-5-methoxy-7-methyl-1H-indole-1-carboxylate

To a solution of compound **15** (5.0 g, 16.7 mmol), **SM2** (7.2 g, 25.1 mmol), and Ir (CO)_2_Acac (0.25 g, 0.05% M-21) placed in the reactor was added anhydrous ethanol (20 mL). After adding DIPEA (2.4 g, 18.4 mmol), the solution was vacuumed with three hydrogen displacements, the internal pressure of 20 bar was maintained, and then the external temperature was raised to 90 °C. The internal pressure reached 24 bar for 14 h after stabilization, and the internal temperature was reduced to ambient temperature while the internal pressure was decreased to normal pressure. After the reaction was monitored by TLC (PE: EA = 5:1, PE: EA = 3:1 R_f_ = 0.4), a colorless oil weighing 8.5 g was obtained by dichloromethane extraction and washed with saturated salt water with vacuum concentration. Crude yield 95%; MS(ESI) *m*/*z*: [ M + H] ^+^ 537.29, found 537.29. ^1^H NMR(600 MHz, CDCl3) δ 8.03 (d, *J* = 7.8 Hz, 2H), 7.63 (d, *J* = 7.4 Hz, 2H), 7.47 (d, *J* = 3.2 Hz, 1 h), 6.66 (s, 2H), 3.91 (s, 3H), 3.79 (s, 3H), 3.60 (dd, *J* = 26.9, 11.9 Hz, 3H), 3.49 (q, *J* = 6.9 Hz, 3H), 3.30 (d, *J* = 12.2 Hz, 1H), 2.63 2.55 (m, 4H), 2.41 (s, 1H), 1.94 (d, *J* = 13.9 Hz, 1H), 1.77 (d, *J* = 12.6 Hz, 2H),1.61 (s, 9H), 1.25 (t, *J* = 6.9 Hz, 3H).

### 4.10. Synthesis of Iptacopan Hydrochloride (LNP023)

Compound **9** (8.5 g, 15.8 mmol) was placed in a 100 mL single-mouth bottle; methanol (20 mL) and tetrahydrofuran (10 mL) were added to the reaction bottle at an external temperature of 45 °C, and 1 M LiOH (79 mL, 79 mmol) aqueous solution was added in batches. After the thermal insulation reaction, TLC was monitored (DCM:MeOH = 10:1 R_f_ = 0.5). Upon completion of the reaction, the concentration was decompressed, then 10% acetonitrile/water was added to dissolve the samples using wet reverse-phase column chromatography (phase A: 1%HCl; B phase: acetonitrile), which eventually obtained white solid powder weighing 4.34 g; yield 60%; mp. 205.5–207 °C; HRMS(ESI) *m*/*z*: calculated for C_25_H_30_N_2_O_4_^+^ [M + H]^+^ 423.22746, found 423.22746. ^1^H NMR(600 MHz, MeOD) δ 8.20 (d, *J* = 8.1Hz, 2H), 7.72 (d, *J* = 7.7 Hz, 2H), 7.32 (s, 1 H), 6.76 (s, 1 H), 6.36 (s, 1 H), 4.78 (d, *J* = 7.9 Hz, 1 h), 4.28 (dd, *J* = 53.9, 12.8 Hz, 2H), 3.82 (s, 1 H), 3.76 (s, 3 H), 3.64 3.52 (m, 3H), 3.35 (s, 1H), 2.50 (s, 3H), 2.25 (d, *J* = 6.4 Hz, 2H), 2.12–1.94 (m, 3H), 1.31 (t, *J* = 6.8 Hz, 3H). ^13^C NMR (101 MHz, MeOD) δ 152.6 (s), 139.6 (s), 134.2 (s), 131.0 (s), 130.2 (s), 129.4 (s), 128.0 (s), 126.9 (s), 124.3 (s), 107.1 (s), 103.5 (s), 98.7 (s), 68.8 (s), 63.9 (s), 63.7 (s), 55.2 (s), 51.7 (s), 48.8 (s), 36.2 (s), 26.3 (s), 15.9 (s), 14.5 (s).

## Figures and Tables

**Table 1:** Effects of temperature, time, and the reaction solvent on reaction ^a^.

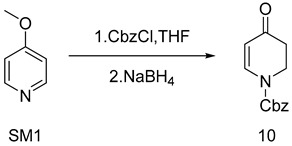				
Entry	Solvent	Temperature ^b^ (°C)	Reaction Time (h)	Yield ^c^ (%)
1	MeOH/THF (5/1)	−78	3	41
2	THF	−78	3.5	50
3	THF	−78	4.5	60
4	THF	−78	7.5	62
5	THF	−78	4.5	63
6	THF	−20	2	85
7	THF	0	2	51
8	THF	25	2	23

^a^ Reaction conditions: all experiments were carried out on a 10 g scale of **SM1**; The sodium borohydride dosage of all experiments was 1.05, equivalent of **SM1**. ^b^ Measurement of temperature: Use a calibrated Celsius thermometer. ^c^ Calculated by the quality of the product actually obtained.

**Table 2:** Effect of reaction conditions on the synthesis of compound **11** ^a^.

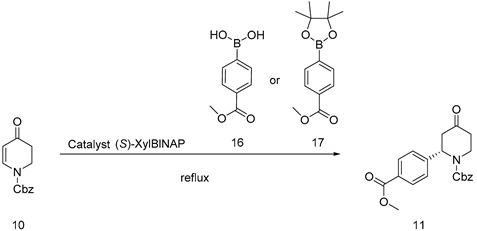					
Entry	16/17(Equiv.)	Catalyst(Equiv.)	Base(Equiv.)	Solvent(Proportion)	Yield
1	16(3)	Rh(Acac)(C_2_H_5_)_2_(0.03)	-	2-Methyl-2-butanol:H_2_O = 10:1	-
2	16(3)	Rh(Acac)(C_2_H_5_)_2_(0.03)	Cs_2_CO_3_(3)	1,4-Dioxane:H_2_O = 5:1	15%
3	17(4)	Pd(OAc)_2_(0.03)	Cs_2_CO_3_(3)	1,4-Dioxane:H_2_O = 5:1	-
4	17(4)	Pd[P(C_6_H_5_)_3_]_4_(0.03)	Cs_2_CO_3_(3)	1,4-Dioxane:H_2_O = 5:1	-
5	17(4)	Pd(C_5_H_7_O_2_)_2_(0.03)	Cs_2_CO_3_(3)	1,4-Dioxane:H_2_O = 5:1	-
6	17(2)	Rh(Acac)(C_2_H_5_)_2_(0.03)	Cs_2_CO_3_(3)	1,4-Dioxane:H_2_O = 5:1	33%
7	17(2.5)	Rh(Acac)(C_2_H_5_)_2_(0.03)	Cs_2_CO_3_(3)	1,4-Dioxane:H_2_O = 5:1	79%
8	17(3)	Rh(Acac)(C_2_H_5_)_2_(0.03)	Cs_2_CO_3_(3)	1,4-Dioxane:H_2_O = 5:1	80%
9	17(4)	Rh(Acac)(C_2_H_5_)_2_(0.03)	Cs_2_CO_3_(3)	1,4-Dioxane:H_2_O = 5:1	81%
10	17(4)	Rh(Acac)(C_2_H_5_)_2_(0.03)	Cs_2_CO_3_(3)	1,4-Dioxane	-
11	17(4)	Rh(Acac)(C_2_H_5_)_2_(0.03)	Cs_2_CO_3_(3)	1,4-Dioxane:H_2_O = 10:1	36%
12	17(4)	Rh(Acac)(C_2_H_5_)_2_(0.03)	Cs_2_CO_3_(3)	1,4-Dioxane:H_2_O = 20:1	34%
13	17(4)	Rh(Acac)(C_2_H_5_)_2_(0.03)	Cs_2_CO_3_(3)	Tol:H_2_O = 5:1	85%
14	17(4)	Rh(Acac)(C_2_H_5_)_2_(0.03)	Cs_2_CO_3_(3)	ACN:H_2_O = 5:1	25%
15	17(4)	Rh(Acac)(C_2_H_5_)_2_(0.03)	Cs_2_CO_3_(3)	THF:H_2_O = 5:1	50%
16	17(4)	Rh(Acac)(C_2_H_5_)_2_(0.03)	TEA(3)	1,4-Dioxane:H_2_O = 5:1	-
17	17(4)	Rh(Acac)(C_2_H_5_)_2_(0.03)	Pyridine(3)	1,4-Dioxane:H_2_O = 5:1	-
18	17(4)	Rh(Acac)(C_2_H_5_)_2_(0.03)	K_2_CO_3_	1,4-Dioxane:H_2_O = 5:1	35%

^a^ Reaction conditions: all experiments were carried out on a 10 g scale of **10**; all reactions are reflux; the time of all reactions is 24 h.

**Table 3:** Screening of KREDs (ketoreductase) from Our Enzyme Library.

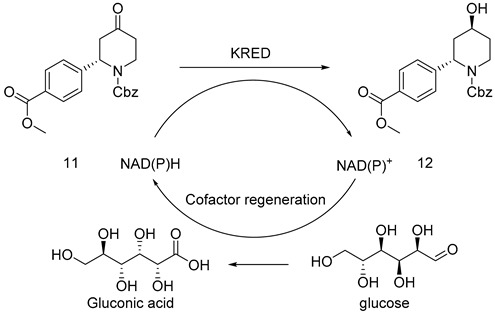			
Entry	Conservation (%)	*ee* ^c^ (%)	Configuration
K2 ^a^	95	72.94	R
K17 ^a^	95	63.74	S
K30 ^a^	80	79.22	S
K31 ^b^	80	99.99	R
K73 ^a^	95	99.99	S

^a^ Reaction conditions: 10 g/L ketone **11**, 5 g/L frozen cell pellets, 0.5 g/L NADP^+^, 2 g/L GDH frozen cell pellets, and 2 equiv. of glucose in 2 mL of PBS buffer (100 mM, pH 7.0) for 24 h at 25 °C and 220 rpm. ^b^ 10 g/L ketone **11**, 5 g/L frozen cell pellets, 0.5 g/L NAD^+^, and 3 equiv. of IPA in 2 mL of PBS buffer (100 mM, pH 7.0) for 24 h at 25 °C and 220 rpm. ^c^ Measured by a chiral IC column with chiral HPLC.

**Table 4:** Comparison of chemical induction and enzymatic induction of 12 synthesis ^a^.

Entry	KRED-M8	(S) CBS Catalyst
Solvent	water/DMSO	THF
Catalyst loading	25 g/L	1.2 eq
Catalyst cost	¥125	¥7840
Co-factor b ^b^ cost	¥66	0
Yield	92%	65%
Purity	99.50%	>95%
*ee*	99.99%	32%

^a^ The data were calculated by catalyzing 1 kg of substrate. ^b^ Refers to NADP^+^.

**Table 5:** Optimization synthesis of compound **14** ^a^.

^ 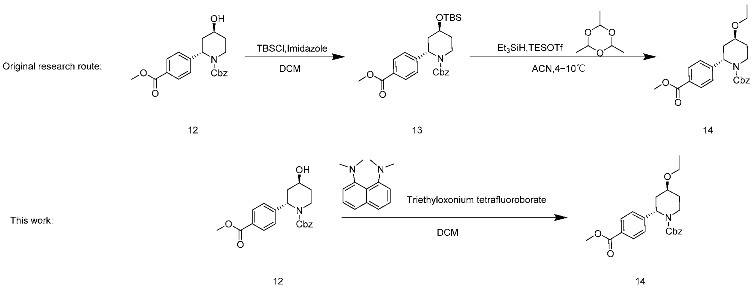 ^			
Entry	Alkylating Reagent	Conversion (%)	Yield (%)
1	N-ethylpyridinium tetrafluoroborate	0	-
2	Trifluoromethanesulfonic anhydride/EtOH	0	-
3	Sodium hydrogen/Iodoethane	0	-
4	Triethyloxonium tetrafluoroborate	99.9	75
5	Paraldehyde	83.0	44
6	Triethyloxonium tetrafluoroborate (No added proton sponge)	48.5	-
7	Triethyloxonium tetrafluoroborate (Added base)	50.3	-

^a^ reaction condition: all experiments were conducted on a 0.5 g scale of **12** or **13**.

## Data Availability

Data are contained within the article and Supplementary Materials.

## Supplementary Materials

The following supporting information can be downloaded at: https://www.mdpi.com/article/10.3390/molecules29102289/s1, WTEA Variants and ^1^H NMR, HRMS,^13^CNMR spectra of **10**, **11**, **12**, **14**, **15**, and **LNP023**; MS, ^1^H NMR spectra of **9**; and ^13^C NMR spectra of **15** and chiral HPLC chromatograms for **12** and chiral isomers; single crystal data of **15** (PDF). Figure S1: MS,^1^H NMR, ^13^C NMR data of 10 and MS data of 22; Figure S2: MS,^1^H NMR, ^13^C NMR data of 11 and MS data of 23; Figure S3: MS,^1^H NMR, ^13^C NMR data of 12; Figure S4: MS, ^1^H NMR, ^13^C NMR data of 14; Figure S5: MS, ^1^H NMR, ^13^C NMR data of 15; Figure S6: MS, ^1^H NMR data of 9; Figure S7: MS, ^1^H NMR, ^13^C NMR data of LNP023; Figure S8: Chiral HPLC chromatogram of compound rac-11 and 11; Figure S9: Chiral HPLC chromatogram of compound rac-12 and 12; Table S1:WTEA Variants and the effect was compared with that of the wild-type and mutant M8; Table S2: Crystal data and structure refinement for 15; Table S3: Atomic coordinates (×10^4^) and equivalent isotropic displacement parameters (A^2^ × 10^3^) for 15; Table S4: Bond lengths [A] and angles [deg] for 15; Table S5: Torsion angles [deg] for 15; Table S6: Hydrogen bonds for 15 [A and deg].
