# Supplementary material for: A Practical Method for Synthesizing Iptacopan
Source: Molecules. 2024 May 13;29(10):2289. doi: 10.3390/molecules29102289 (PMC11124358; doi:10.3390/molecules29102289)
Supplement: Supplementary file 1 [file molecules-29-02289-s001.zip › molecules-2982838-supplementary.pdf]

Supporting information

A practical method for synthesizing Iptacopan

Zhiwei Tang,[1] Shaojie Chu,[1] Xuesong Wu,[1] Shaoxin Chen,[1] Likuo Chen,[1] Jiawei Tang,[1]* and Hongbo Wang[1]*

[1] National Key Laboratory of Lead Druggability Research, Shanghai Institute of Pharmaceutical Industry, China State Institute of Pharmaceutical Industry, 285 Gebaini Road, Pudong, Shanghai 201203, China

Table of Contents

1.Table S1. WTEA Variants and the effect was compared with that of the wild-type and mutant M8 2

2.Figure S1. MS,^{1}H NMR, ^{13}C NMR data of 10 and MS data of 22 3

3.Figure S2. MS,^{1}H NMR, ^{13}C NMR data of 11 and MS data of 23 4

4.Figure S3. MS,^{1}H NMR, ^{13}C NMR data of 12 7

5.Figure S4. MS, ^{1}H NMR, ^{13}C NMR data of 14 8

6.Figure S5. MS, ^{1}H NMR, ^{13}C NMR data of 15 10

7.Figure S6. MS, ^{1}H NMR data of 9 11

8.Figure S7. MS, ^{1}H NMR, ^{13}C NMR data of LNP023 12

9. Figure S8. Chiral HPLC chromatogram of compound rac-11 and 11 13

10. Figure S9. Chiral HPLC chromatogram of compound rac-12 and 12 14

11.Table S2. Crystal data and structure refinement for 15. 15

12.Table S3. Atomic coordinates (x 10^4) and equivalent isotropic displacement parameters (A^2 x 10^3) for 15. 16

13.Table S4. Bond lengths [A] and angles [deg] for 15. 17

14.Table S5. Torsion angles [deg] for 15. 19

15.Table S6. Hydrogen bonds for 15 [A and deg.] 20

1.**Table S1. WTEA Variants and the effect was compared with that of the wild-type and mutant M8**

Enzyme	Mutated residues
M1	W82L/V121A/A138L/A190V/S193A/K206H
M2	W82L/F88V/V121A/A138L/A190V/S193A/K206H/K207N
M3	F88V/V121A/A138L/R142M/A190V/S193A
M4	T17V/W82L/F88V/V121A/A138L/R142M/A190V/S193A/Y201F/N204A
M5	W82L/F88V/V121A/A138L/R142M/A190V/S193A/Y201F/N204A
M6	F88I/A138V/R142M/A190V/S193A
M7	F88V/A138L/R142M/A190V/S193A/N204A
M8	W82L/F88V/V121A/A138L/R142M/A190V/S193A

Table S1a. The effect was compared with that of the wild-type and mutant M8

**mutant**	**Amino acid residue differences from SEQ ID NO:1**	**Conversion (%)**	**S configuration *ee* value (%)**	**Reaction time (h)**
	wild type	84%	100%	24
M8	W82L/F88V/V121A/A138L/R142M/A190V/ S193A	92%	100%	4

2.Figure S1. MS,^{1}H NMR, ^{13}C NMR data of 10 and MS data of 22

MS spectrum of compound 10

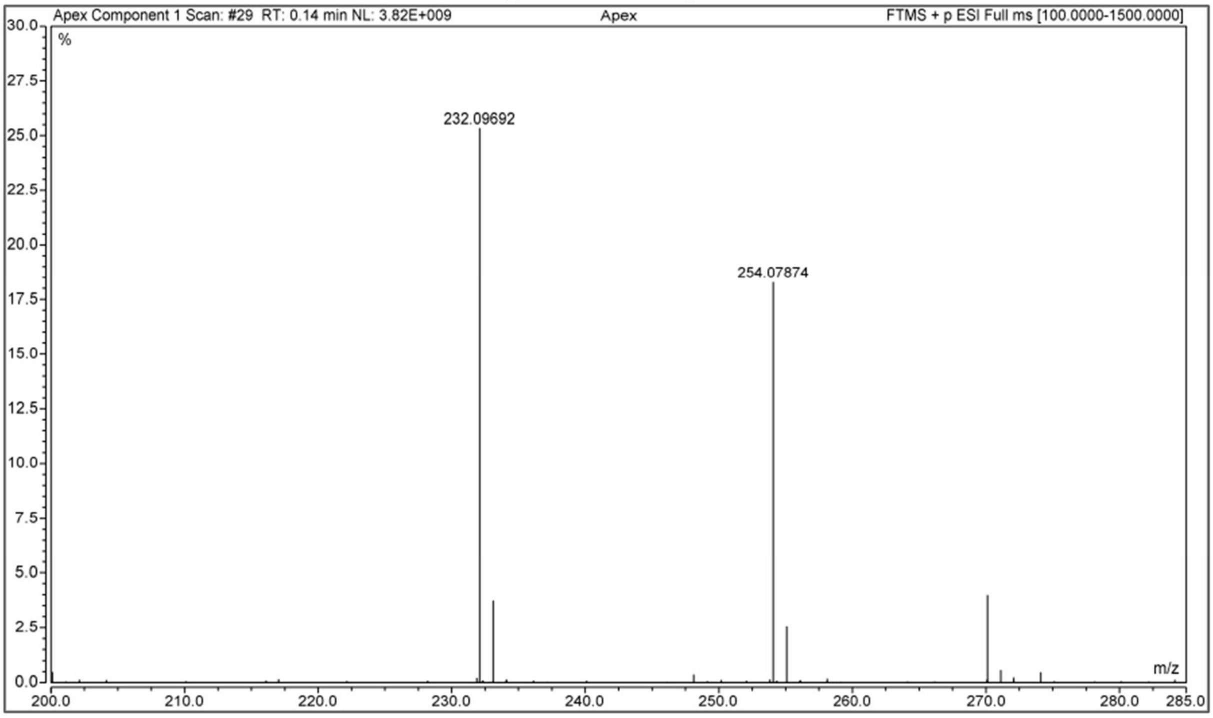

Mass spectrum Peak List						
RT (min)	m/z	Theo. Mass	Charge	Delta (ppm)	Ion	Formula
0.14	232.09692	232.09682	1	0.43	[M+H]+	C13H13NO3
0.14	254.07874	254.07876	1	-0.10	[M+Na]+	C13H13NO3

^{1}H NMR spectrum of compound 10

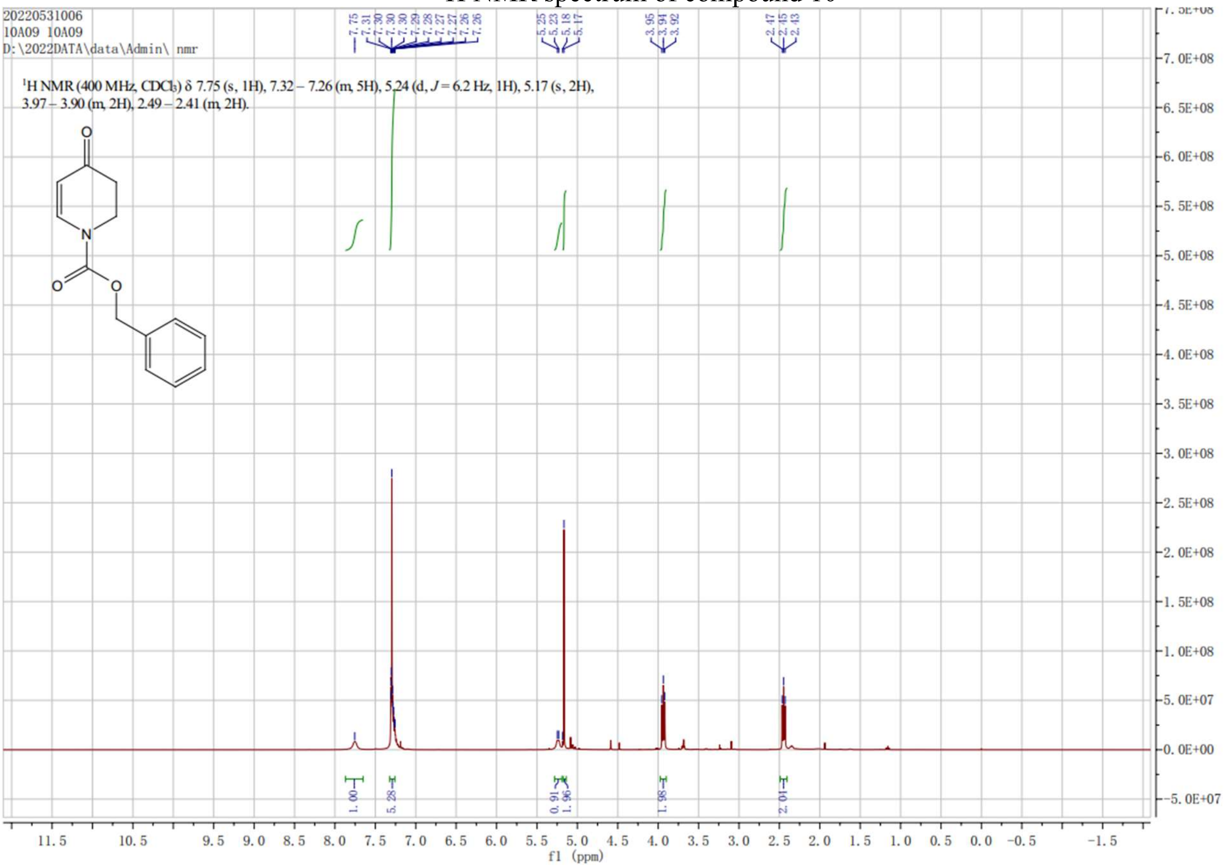

^{13}C NMR spectrum of compound 10

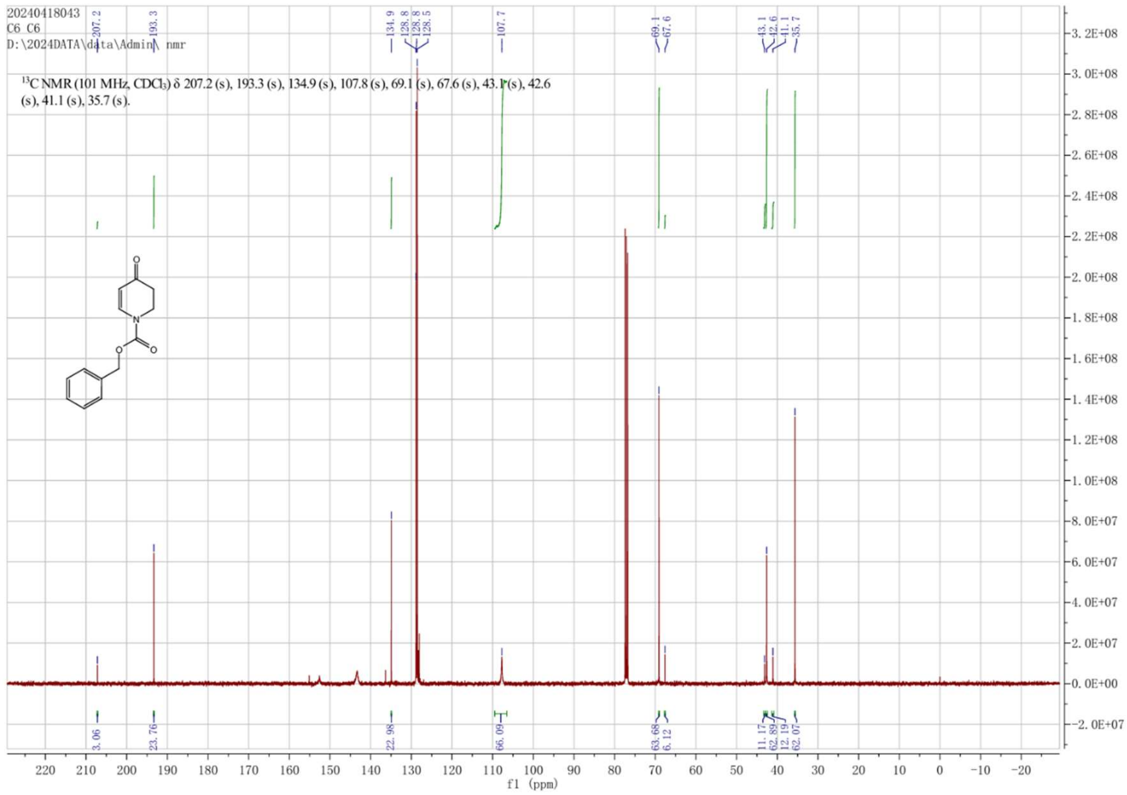

MS spectrum of compound 22

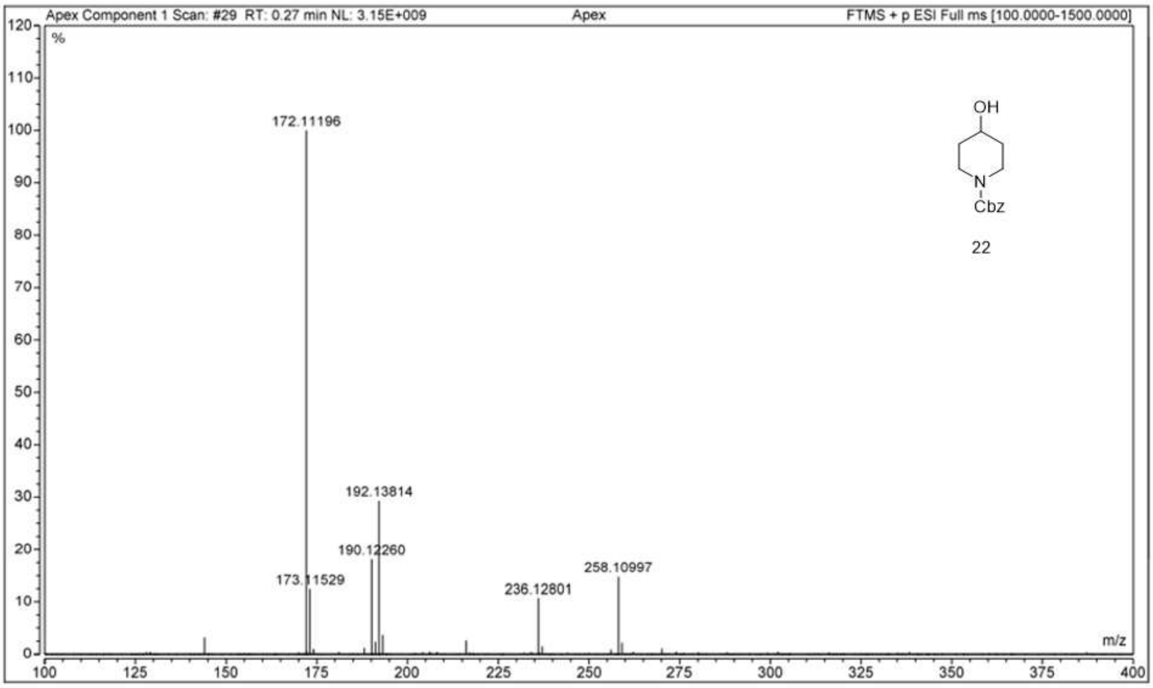

Mass spectrum Peak List						
RT (min)	m/z	Theo. Mass	Charge	Delta (ppm)	Ion	Formula
0.27	236.12801	236.12812	1	-0.47	[M+H]+	C13H17NO3
0.27	258.10997	258.11006	1	-0.37	[M+Na]+	C13H17NO3

3.Figure S2. MS,^{1}H NMR, ^{13}C NMR data of 11 and MS data of 23

MS spectrum of compound 11

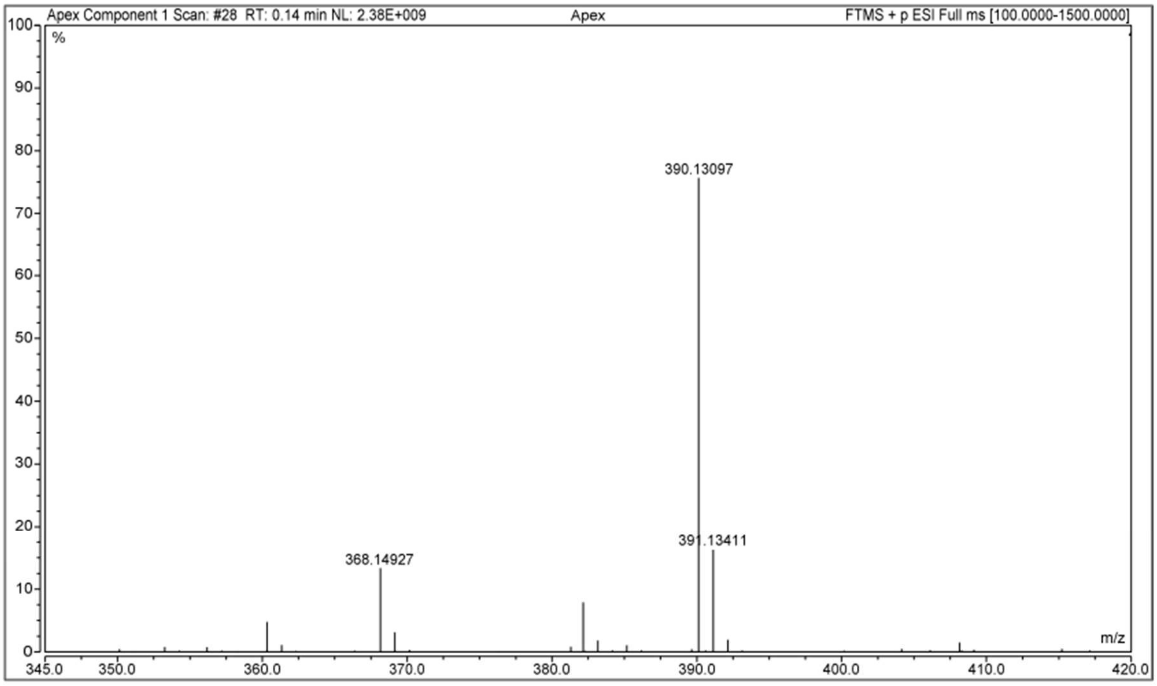

Mass spectrum Peak List						
RT (min)	m/z	Theo. Mass	Charge	Delta (ppm)	Ion	Formula
0.14	368.14927	368.14925	1	0.06	[M+H]+	C21H21NO5
0.14	390.13097	390.13119	1	-0.57	[M+Na]+	C21H21NO5

^{1}H NMR spectrum of compound 11

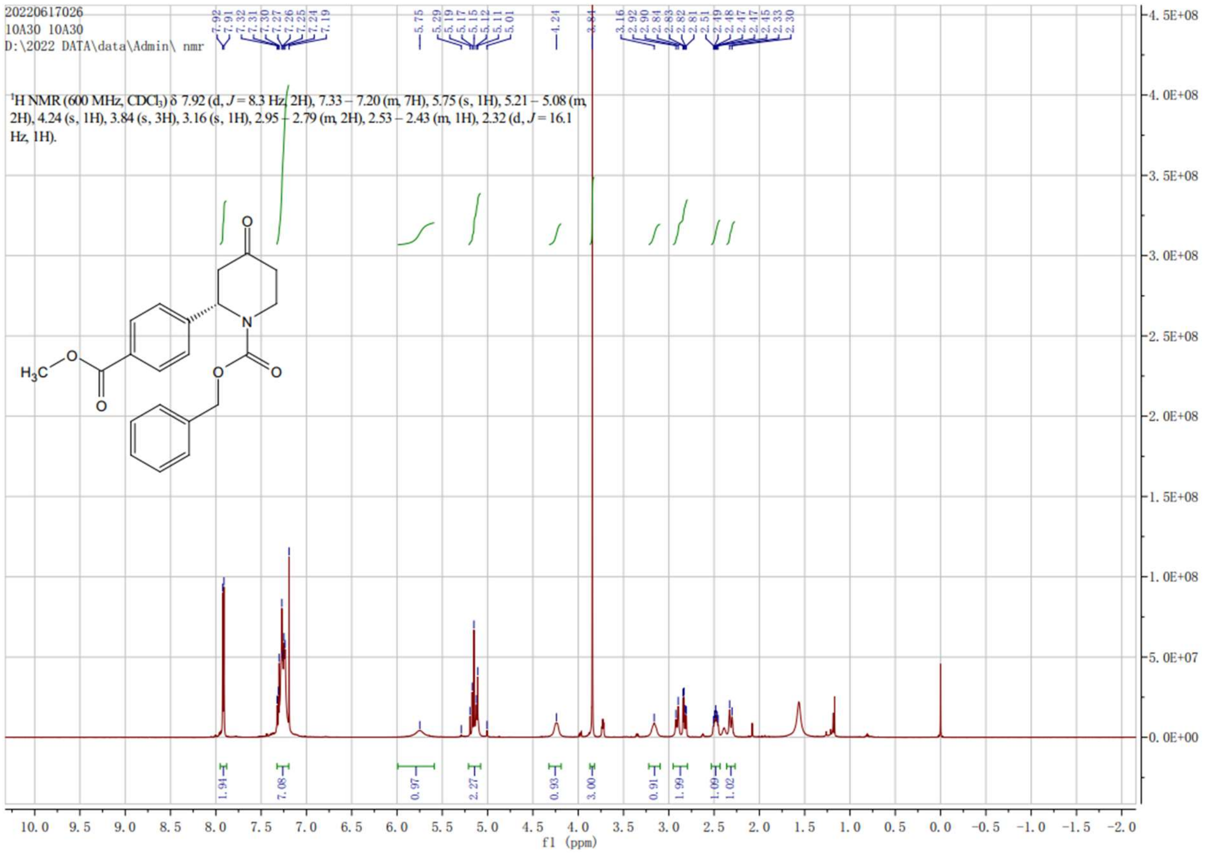

^{13}C NMR spectrum of compound 11

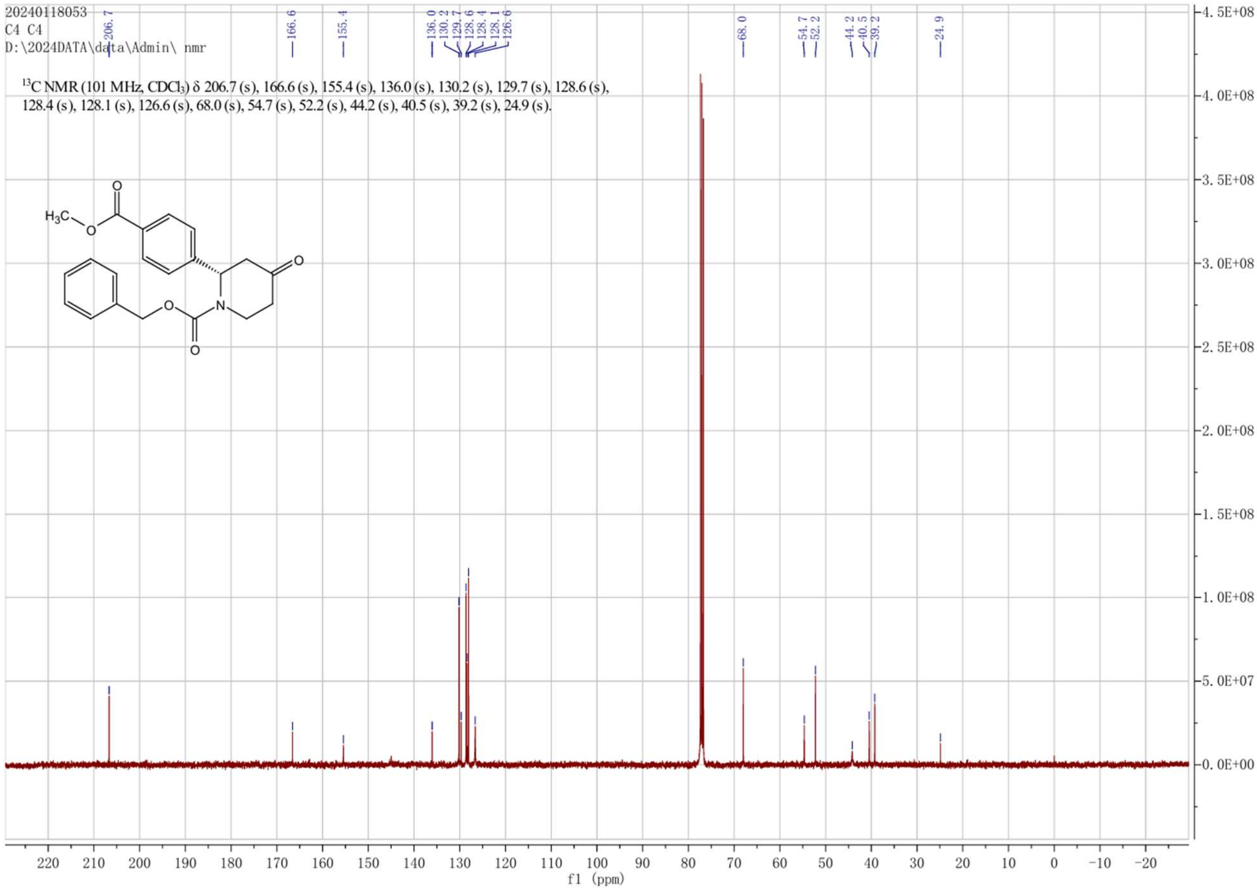

MS spectrum of compound 23

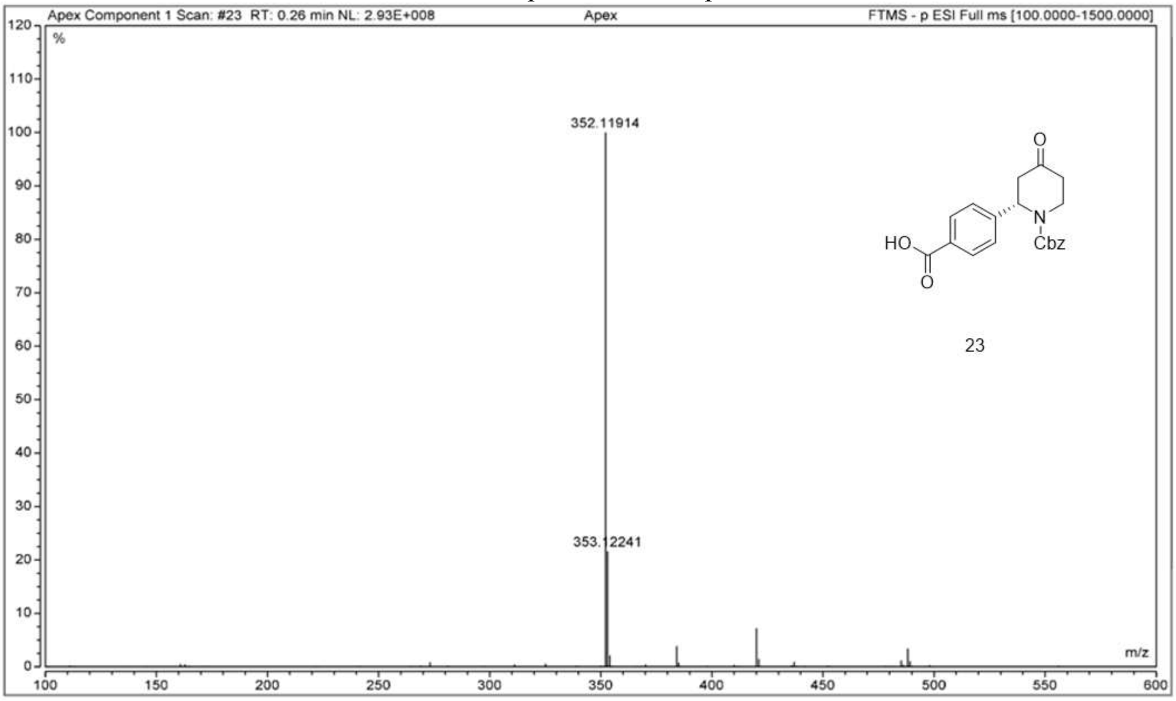

Mass spectrum Peak List						
RT (min)	m/z	Theo. Mass	Charge	Delta (ppm)	Ion	Formula
0.26	352.11914	352.11905	1	0.27	[M-H]-	C20H19NO5

4.Figure S3. MS,^{1}H NMR, ^{13}C NMR data of 12

MS spectrum of compound 12

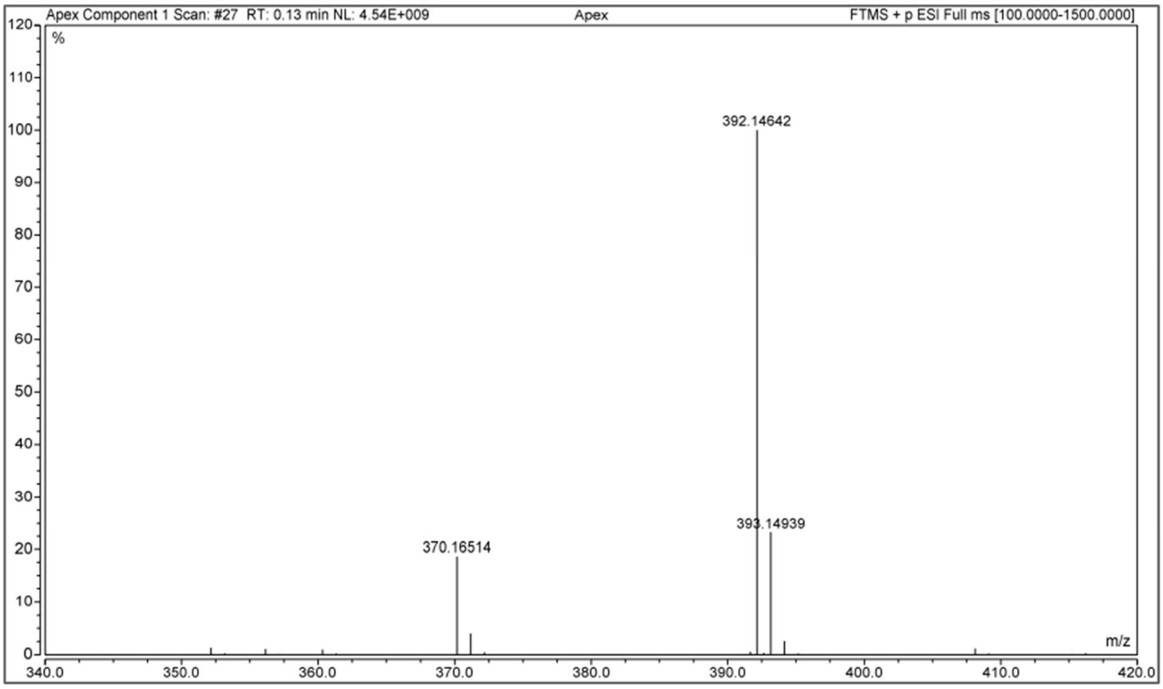

Mass spectrum Peak List						
RT (min)	m/z	Theo. Mass	Charge	Delta (ppm)	Ion	Formula
0.13	370.16514	370.16490	1	0.65	[M+H]+	C21H23NO5
0.13	392.14642	392.14684	1	-1.08	[M+Na]+	C21H23NO5

^{1}H NMR spectrum of compound 12

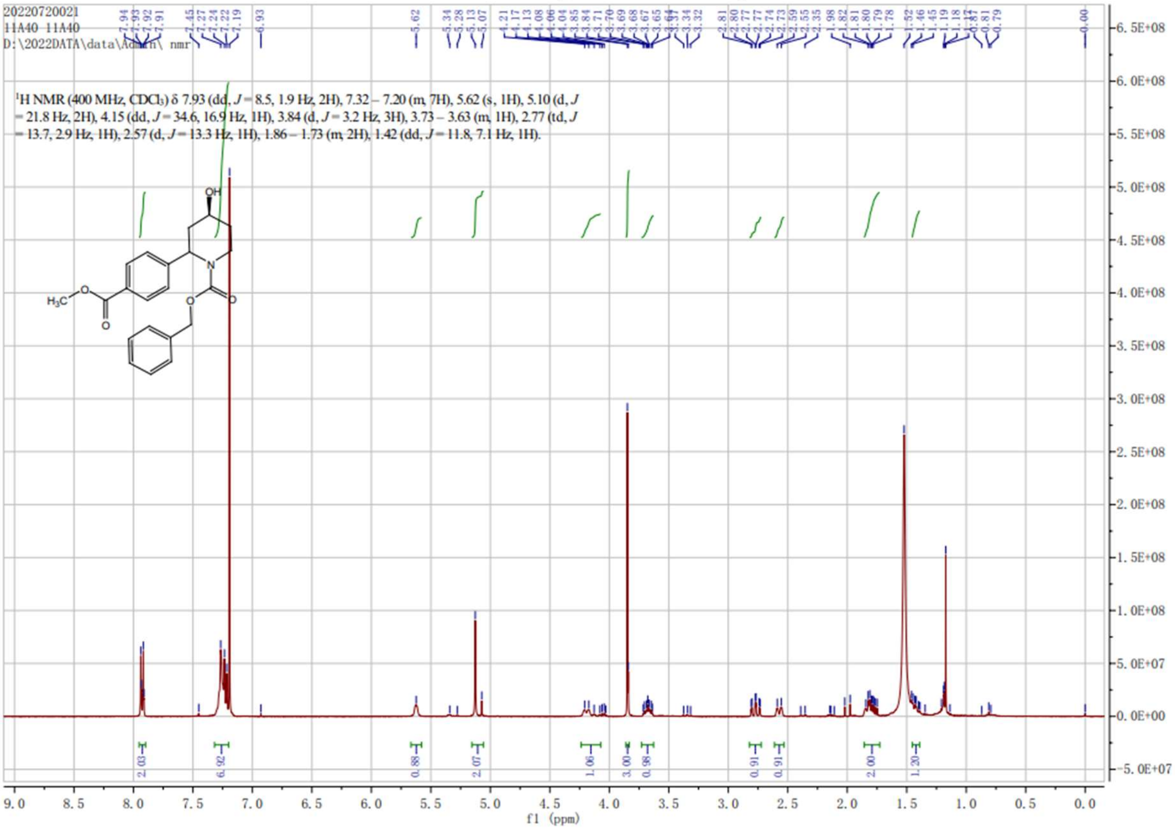

^{13}C NMR spectrum of compound 12

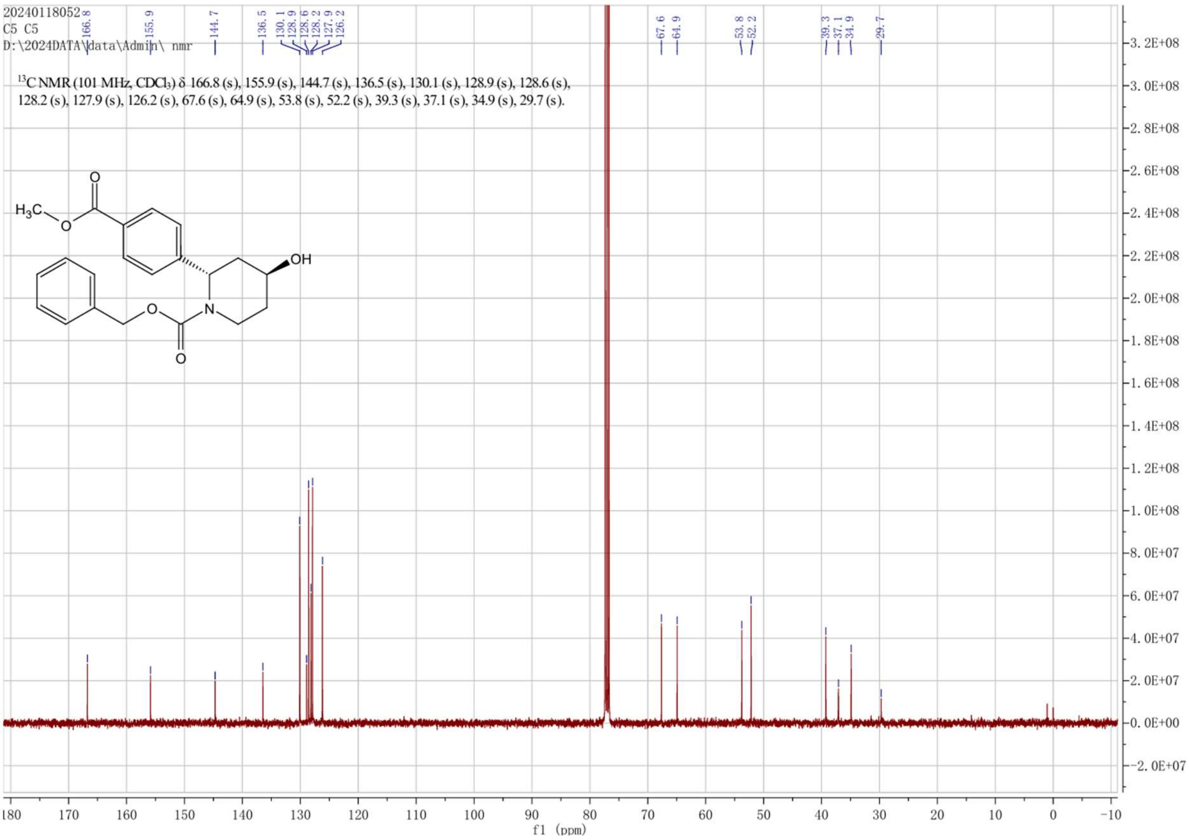

5.Figure S4. MS, ^{1}H NMR, ^{13}C NMR data of 14

MS spectrum of compound 14

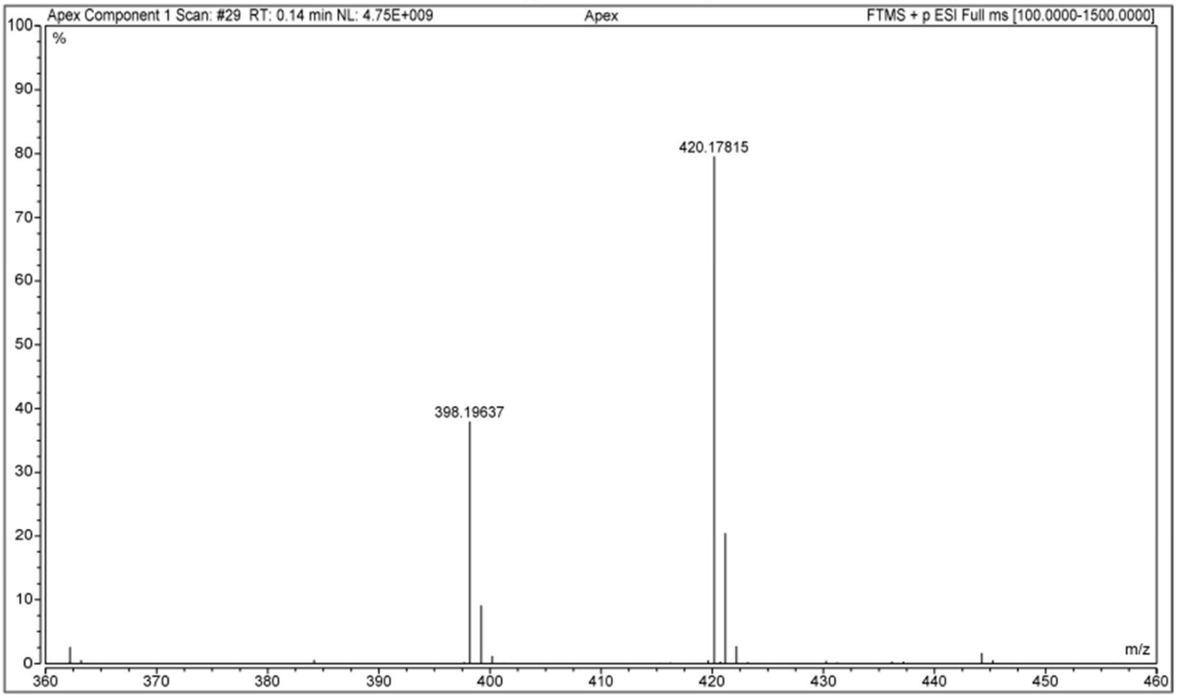

Mass spectrum Peak List						
RT (min)	m/z	Theo. Mass	Charge	Delta (ppm)	Ion	Formula
0.14	398.19637	398.19620	1	0.43	[M+H]+	C23H27NO5
0.14	420.17815	420.17814	1	0.01	[M+Na]+	C23H27NO5

^{1}H NMR spectrum of compound 14

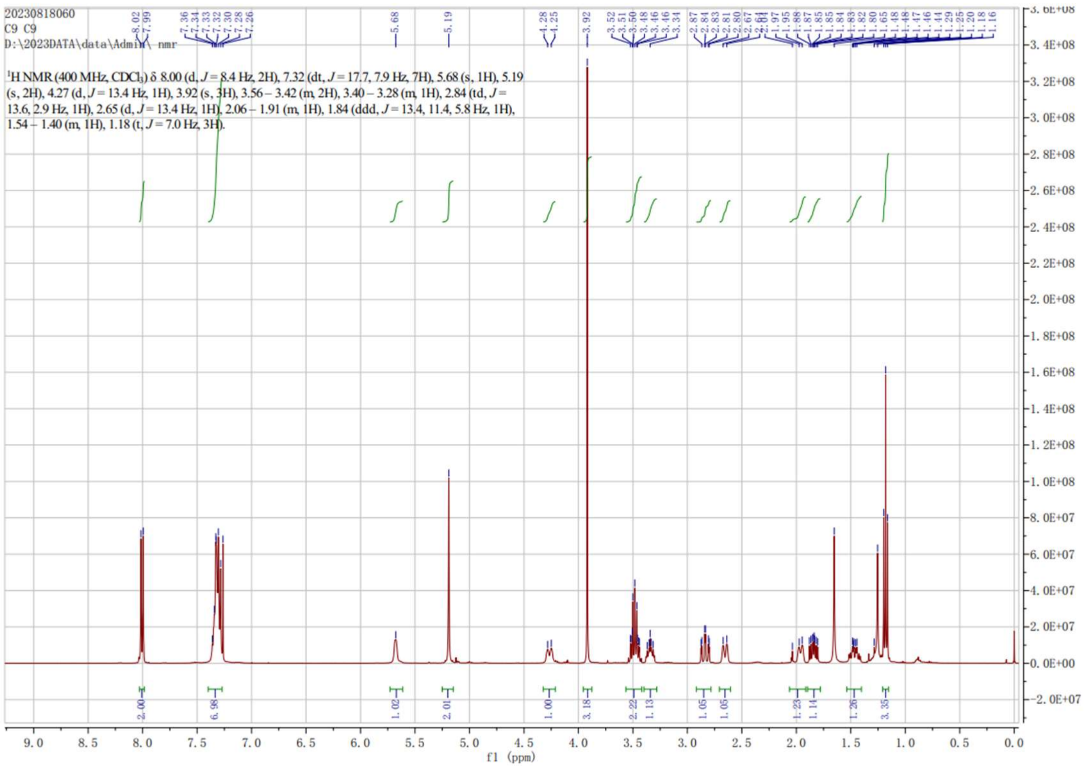

^{13}C NMR spectrum of compound 14

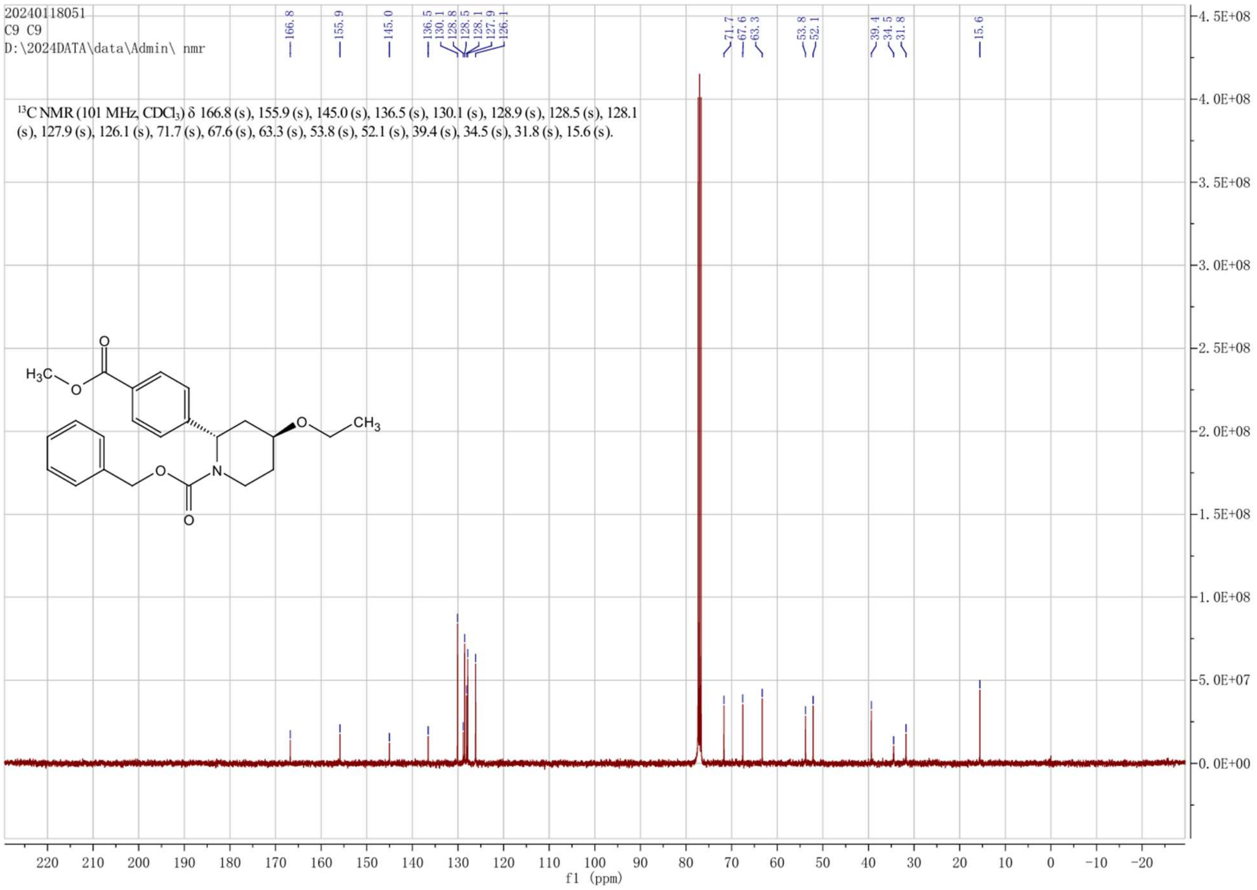

6.Figure S5. MS, ^{1}H NMR, ^{13}C NMR data of 15

MS spectrum of compound 15

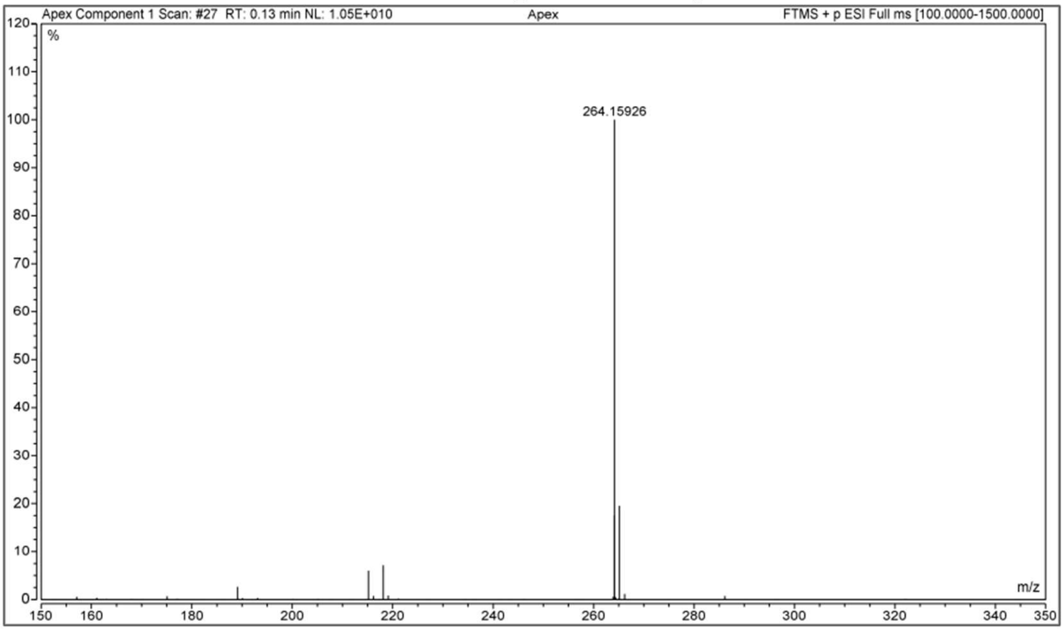

Mass spectrum Peak List						
RT (min)	m/z	Theo. Mass	Charge	Delta (ppm)	Ion	Formula
0.13	264.15926	264.15942	1	-0.61	[M+H]+	C15H21NO3

^{1}H NMR spectrum of compound 15

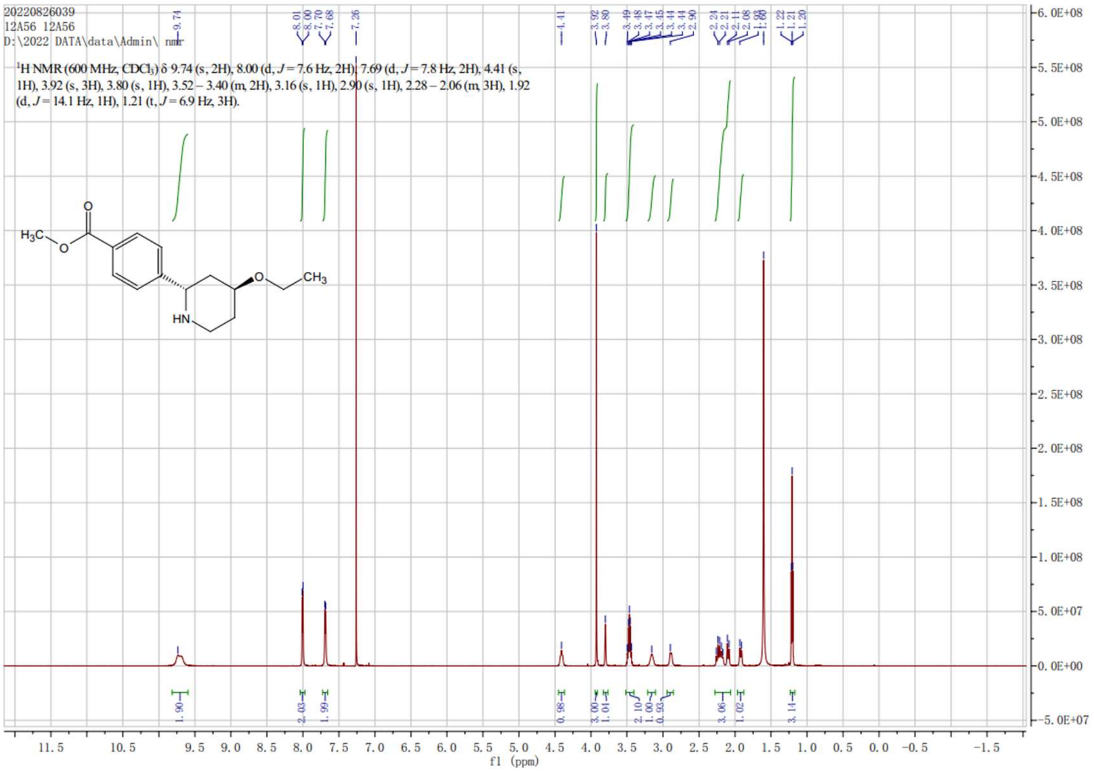

^{13}C NMR spectrum of compound 15

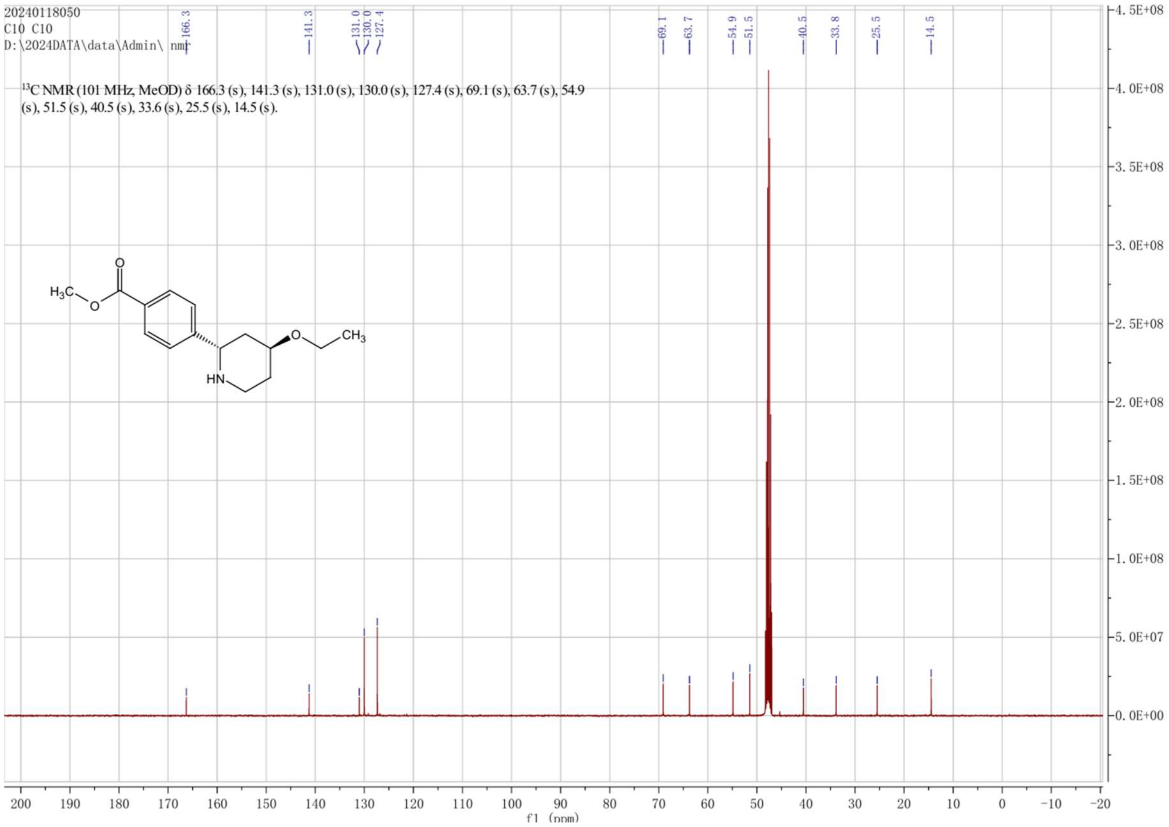

7.Figure S6. MS, ^{1}H NMR data of 9

MS spectrum of compound 9

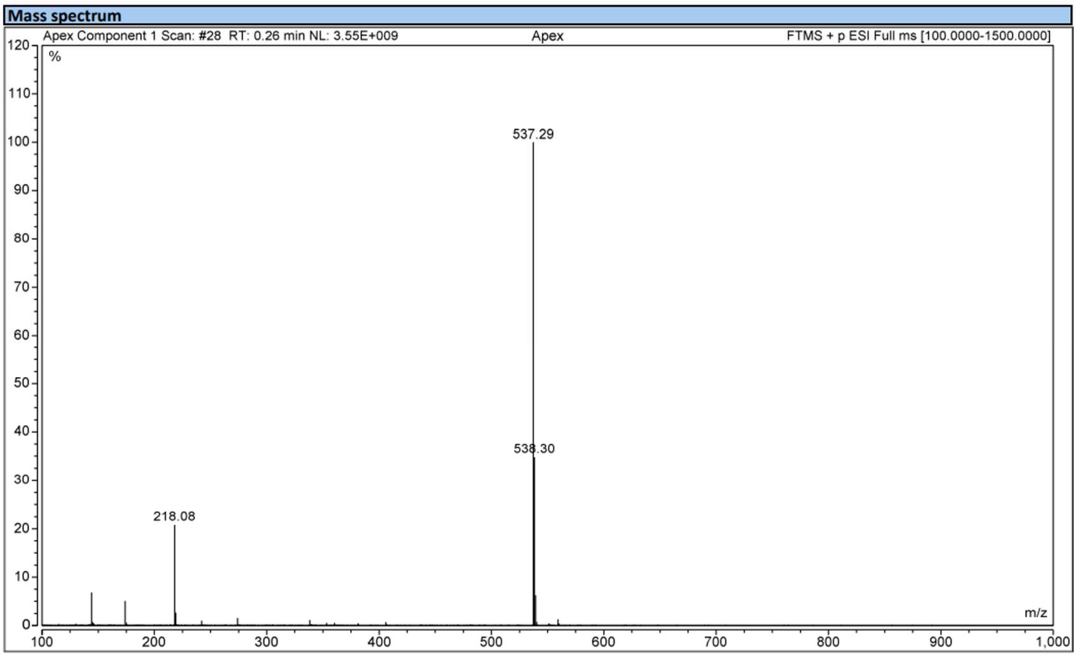

^{1}H NMR spectrum of compound 9

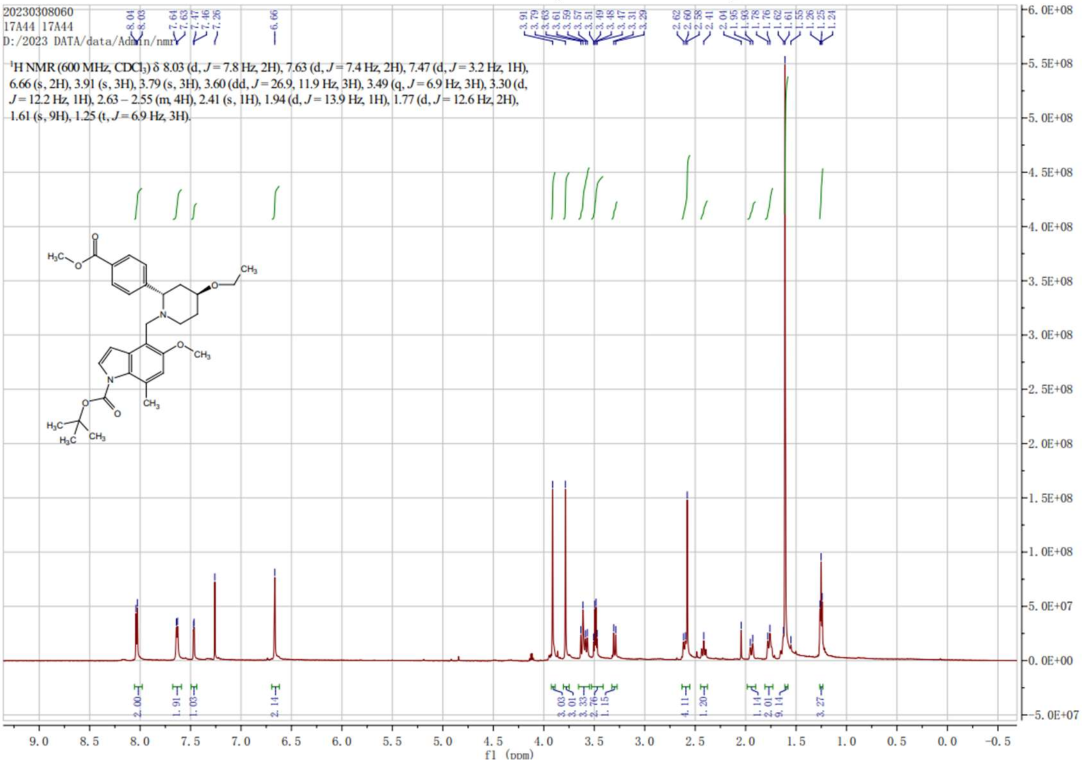

8.Figure S7. MS, ^{1}H NMR, ^{13}C NMR data of LNP023

MS spectrum of LNP023

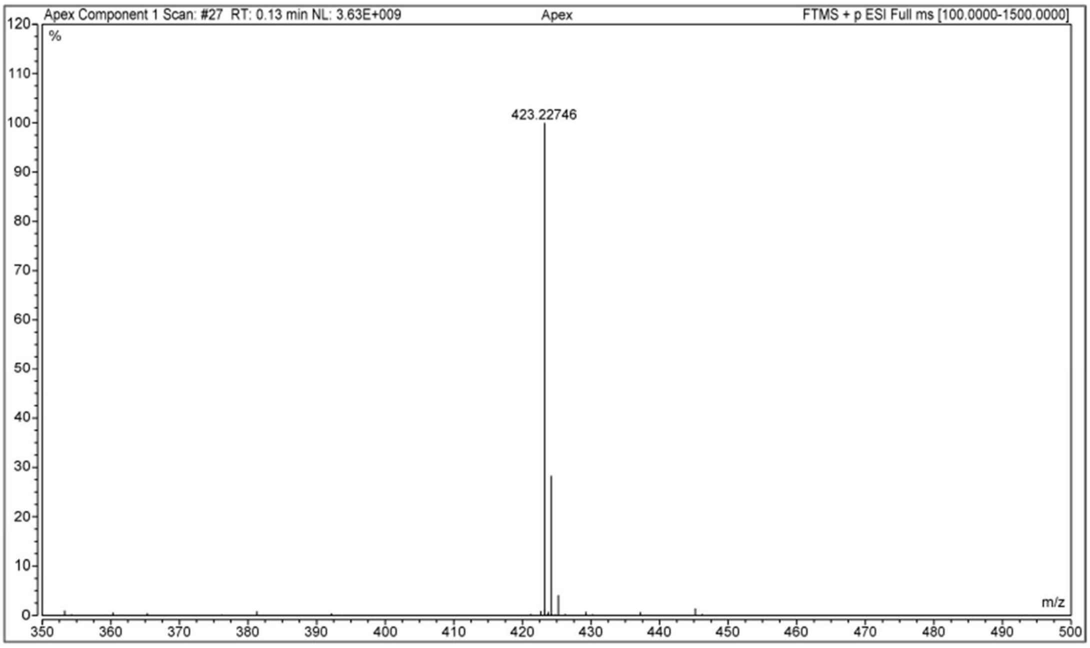

Mass spectrum Peak List						
RT (min)	m/z	Theo. Mass	Charge	Delta (ppm)	Ion	Formula
0.13	423.22746	423.22783	1	-0.88	[M+H]+	C25H30N2O4

^{1}H NMR spectrum of LNP023

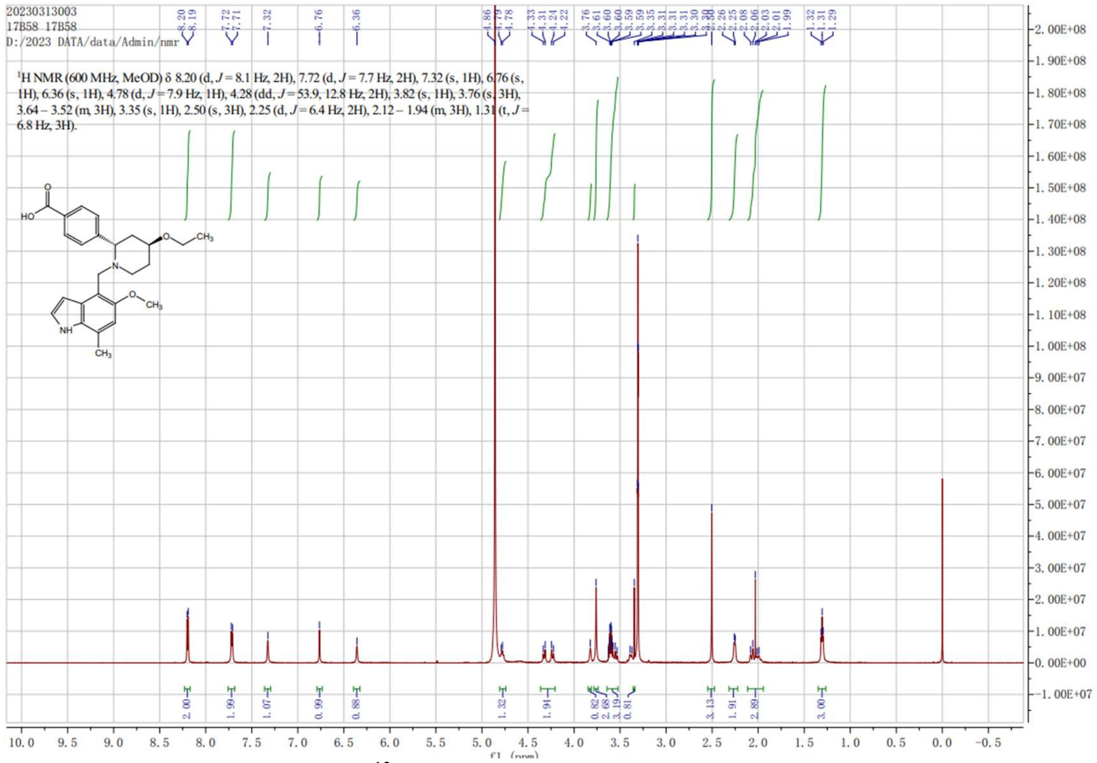

^{13}C NMR spectrum of LNP023

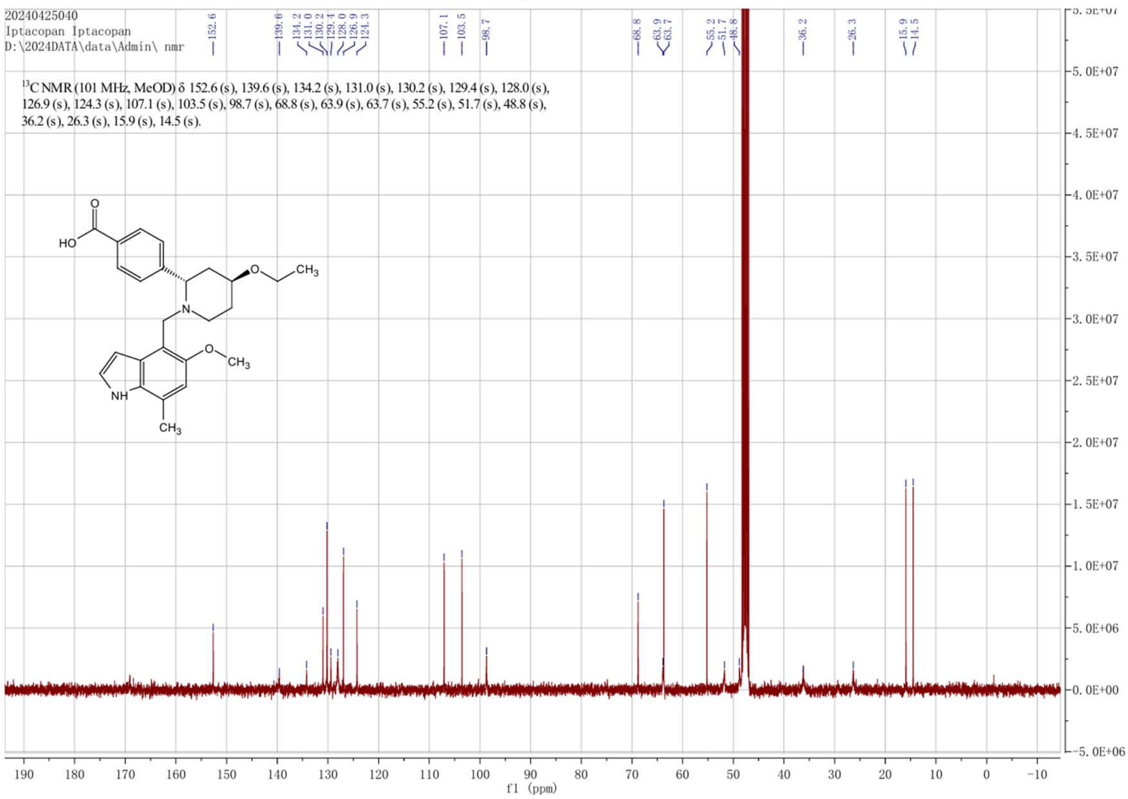

9. Figure S8. Chiral HPLC chromatogram of compound rac-11 and 11

Chiral HPLC chromatogram of compound rac-11

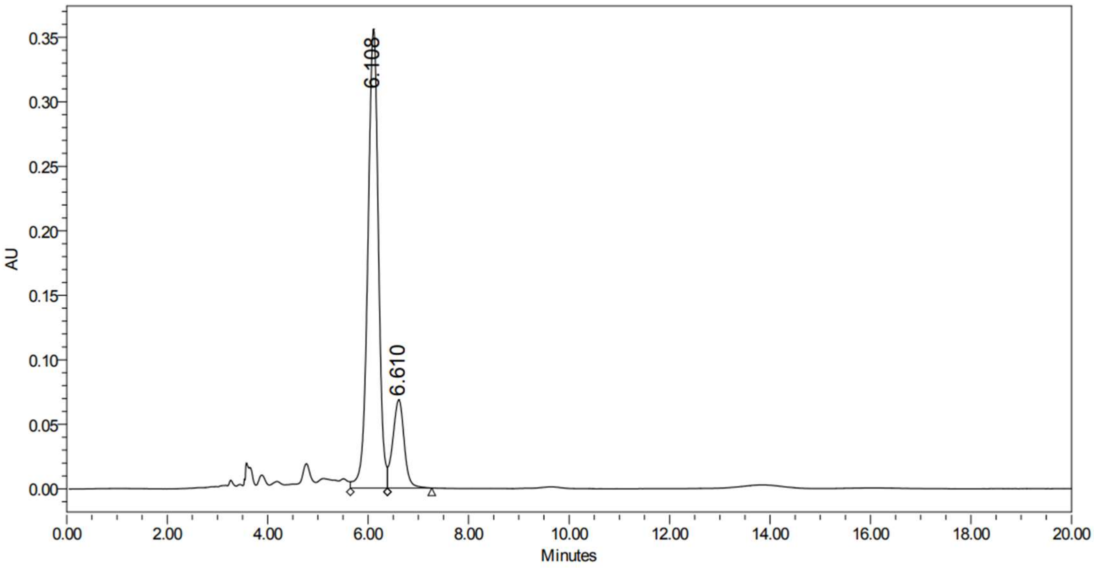

	RT	Area	% Area	Height
1	6.108	5187021	82.76	355801
2	6.610	1080252	17.24	68508

Chiral HPLC chromatogram of compound 11

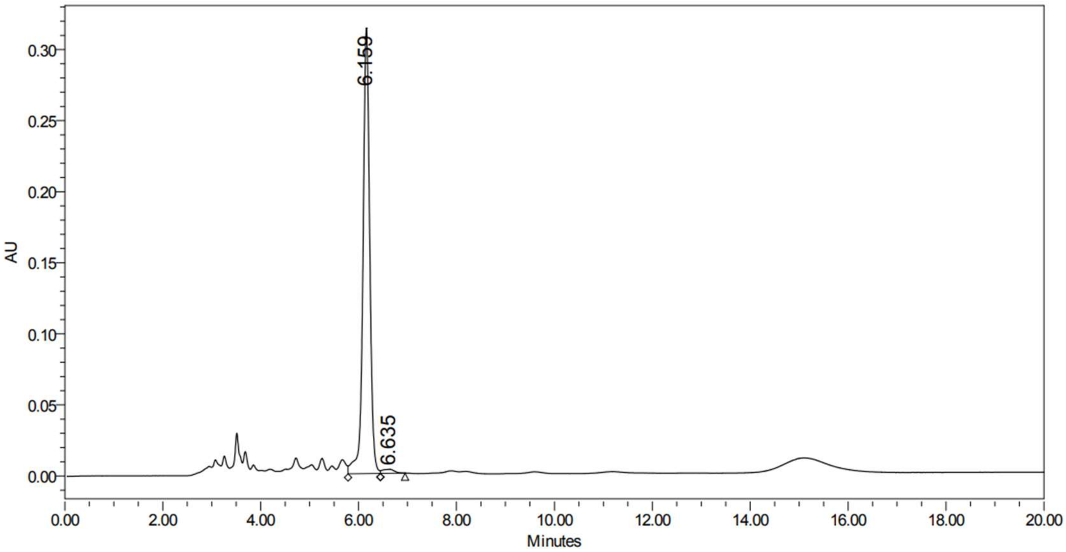

	RT	Area	% Area	Height
1	6.159	2999048	98.42	313489
2	6.635	48116	1.58	2849

10. Figure S9. Chiral HPLC chromatogram of compound rac-12 and 12

Chiral HPLC chromatogram of compound **rac-12**

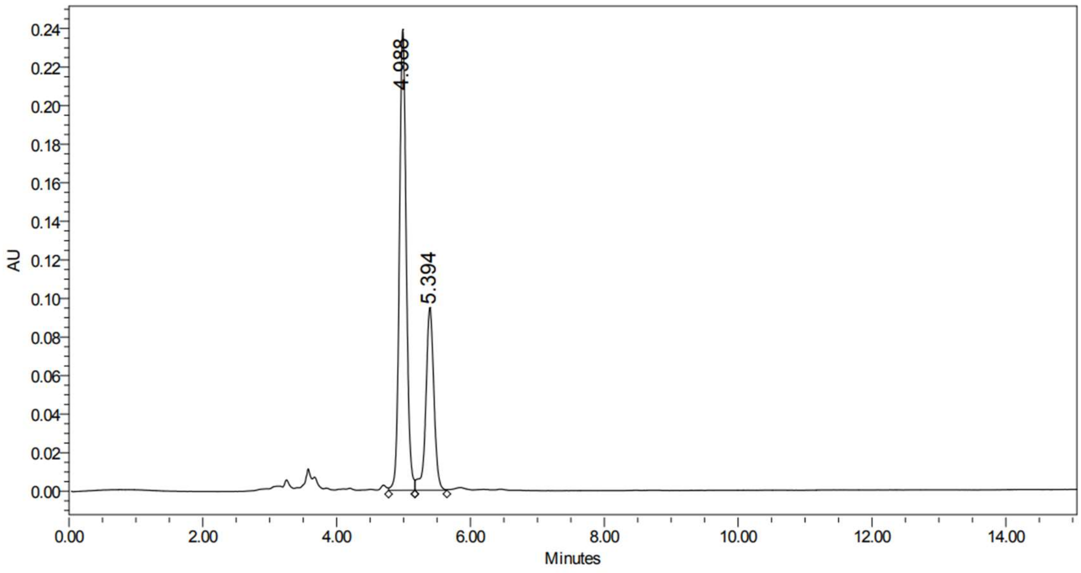

	RT	Area	% Area	Height
1	4.988	1703445	68.35	239144
2	5.394	788778	31.65	94761

Chiral HPLC chromatogram of compound **12**

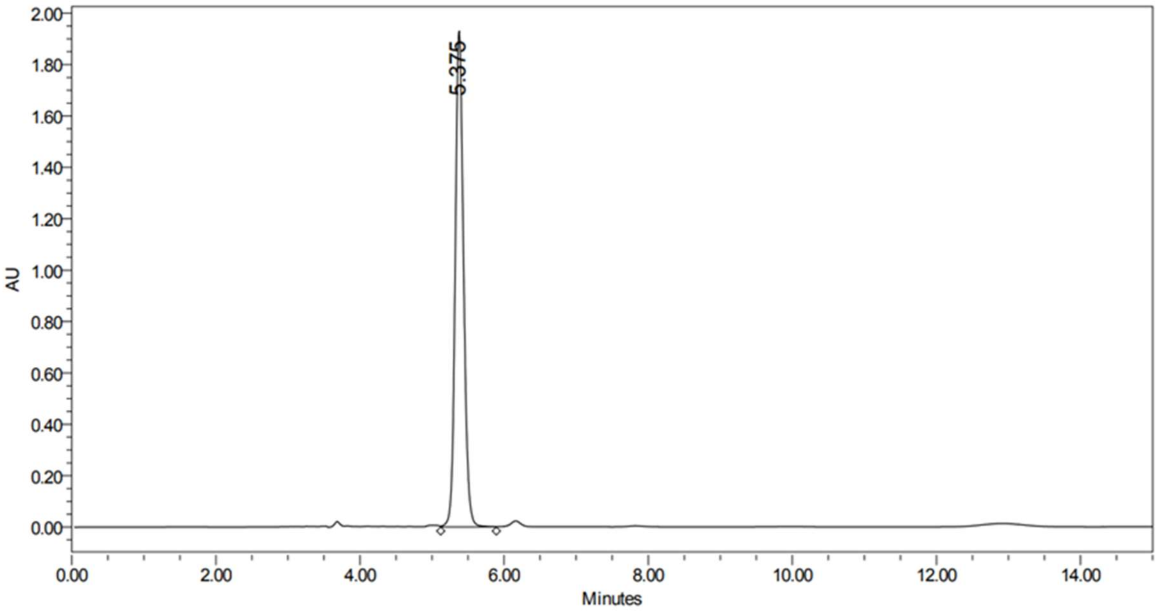

	RT	Area	% Area	Height
1	5.375	15514941	100.00	1928738

11.Table S2. Crystal data and structure refinement for 15.

Identification code S1470_20220905024

Empirical formula	$C_{15}H_{22}ClNO_3$
Formula weight	299.79
Temperature	296(2) K
Wavelength	1.54178 A
Crystal system, space group	Orthorhombic, P2(1)2(1)2(1)
Unit cell dimensions	a = 5.35520(10) A alpha = 90 deg.
	b = 9.75600(10) A beta = 90 deg.
	c = 30.5740(4) A gamma = 90 deg.
Volume	1597.35(4) A^3
Z, Calculated density	4, 1.247 Mg/m^3
Absorption coefficient	2.176 mm^-1
F(000)	640
Crystal size	0.20 x 0.06 x 0.04 mm
Theta range for data collection	2.89 to 68.92 deg.
Limiting indices	-4<=h<=6, -11<=k<=11, -36<=l<=35
Reflections collected / unique	8506 / 2825 [R(int) = 0.0253]
Completeness to theta = 68.92	96.9 %
Absorption correction	Semi-empirical from equivalents
Max. and min. transmission	0.7531 and 0.5904
Refinement method	Full-matrix least-squares on F^2
Data / restraints / parameters	2825 / 0 / 182
Goodness-of-fit on F^2	1.061
Final R indices [I>2sigma(I)]	R1 = 0.0305, wR2 = 0.0843
R indices (all data)	R1 = 0.0316, wR2 = 0.0852
Absolute structure parameter	0.045(13)
Extinction coefficient	0.0060(6)
Largest diff. peak and hole	0.166 and -0.138 e.A^-3

12.Table S3. Atomic coordinates (x 10^4) and equivalent isotropic displacement parameters (A^2 x 10^3) for 15.

	x	y	z	U(eq)
Cl(1)	8977(1)	6815(1)	2100(1)	52(1)
O(1)	13131(3)	4717(2)	-307(1)	82(1)
O(2)	16834(3)	4101(1)	-43(1)	65(1)
O(3)	13932(3)	11674(1)	1520(1)	66(1)
N(1)	13913(3)	8205(1)	1833(1)	44(1)
C(1)	14532(3)	5728(2)	355(1)	46(1)
C(2)	12559(3)	6646(2)	371(1)	54(1)
C(3)	12383(3)	7578(2)	709(1)	52(1)
C(4)	14174(3)	7617(2)	1036(1)	44(1)
C(5)	16118(3)	6668(2)	1025(1)	53(1)
C(6)	16290(3)	5737(2)	686(1)	51(1)
C(7)	14704(4)	4800(2)	-32(1)	54(1)
C(8)	14068(3)	8749(1)	1376(1)	44(1)
C(9)	16297(4)	9716(2)	1349(1)	54(1)
C(10)	16022(4)	10904(2)	1668(1)	59(1)
C(11)	15692(4)	10350(2)	2132(1)	57(1)
C(12)	13578(3)	9347(2)	2159(1)	51(1)
C(13)	17219(6)	3233(2)	-420(1)	79(1)
C(14)	13631(6)	12984(2)	1711(1)	90(1)
C(15)	11785(7)	13787(2)	1453(1)	97(1)
H(1A)	15318	7740	1896	66
H(1B)	12618	7620	1854	66

H(2A)	11349	6634	153	65
H(3A)	11048	8187	717	62
H(5A)	17305	6661	1247	63
H(6A)	17600	5112	680	62
H(8A)	12557	9288	1321	52
H(9A)	17815	9214	1415	65
H(9B)	16438	10071	1053	65
H(10A)	17522	11478	1655	71
H(11A)	17224	9905	2224	69
H(11B)	15374	11107	2330	69
H(12A)	12016	9817	2102	61
H(12B)	13499	8967	2452	61
H(13A)	18807	2783	-396	119
H(13B)	17189	3782	-681	119
H(13C)	15916	2558	-434	119
H(14A)	15221	13460	1715	107
H(14B)	13054	12888	2010	107
H(15A)	11585	14677	1582	146
H(15B)	10208	13318	1453	146
H(15C)	12368	13884	1158	146

13.Table S4. Bond lengths [A] and angles [deg] for 15.

O(1)-C(7)	1.194(2)
O(2)-C(7)	1.329(2)
O(2)-C(13)	1.446(2)
O(3)-C(14)	1.414(2)
O(3)-C(10)	1.422(3)
N(1)-C(8)	1.4985(18)
N(1)-C(12)	1.5047(19)
N(1)-H(1A)	0.9000
N(1)-H(1B)	0.9000
C(1)-C(6)	1.382(2)
C(1)-C(2)	1.386(2)
C(1)-C(7)	1.491(2)
C(2)-C(3)	1.380(2)
C(2)-H(2A)	0.9300
C(3)-C(4)	1.386(2)
C(3)-H(3A)	0.9300
C(4)-C(5)	1.393(2)
C(4)-C(8)	1.517(2)
C(5)-C(6)	1.381(2)
C(5)-H(5A)	0.9300
C(6)-H(6A)	0.9300
C(8)-C(9)	1.524(2)
C(8)-H(8A)	0.9800
C(9)-C(10)	1.522(2)
C(9)-H(9A)	0.9700
C(9)-H(9B)	0.9700
C(10)-C(11)	1.530(2)
C(10)-H(10A)	0.9800
C(11)-C(12)	1.498(2)

C(11)-H(11A)	0.9700
C(11)-H(11B)	0.9700
C(12)-H(12A)	0.9700
C(12)-H(12B)	0.9700
C(13)-H(13A)	0.9600
C(13)-H(13B)	0.9600
C(13)-H(13C)	0.9600
C(14)-C(15)	1.487(4)
C(14)-H(14A)	0.9700
C(14)-H(14B)	0.9700
C(15)-H(15A)	0.9600
C(15)-H(15B)	0.9600
C(15)-H(15C)	0.9600

C(7)-O(2)-C(13)	116.25(16)
C(14)-O(3)-C(10)	115.87(18)
C(8)-N(1)-C(12)	111.26(11)
C(8)-N(1)-H(1A)	109.4
C(12)-N(1)-H(1A)	109.4
C(8)-N(1)-H(1B)	109.4
C(12)-N(1)-H(1B)	109.4
H(1A)-N(1)-H(1B)	108.0
C(6)-C(1)-C(2)	119.24(14)
C(6)-C(1)-C(7)	122.83(15)
C(2)-C(1)-C(7)	117.89(15)
C(3)-C(2)-C(1)	120.29(15)
C(3)-C(2)-H(2A)	119.9
C(1)-C(2)-H(2A)	119.9
C(2)-C(3)-C(4)	120.75(16)
C(2)-C(3)-H(3A)	119.6
C(4)-C(3)-H(3A)	119.6
C(3)-C(4)-C(5)	118.75(14)
C(3)-C(4)-C(8)	119.21(14)
C(5)-C(4)-C(8)	121.89(15)
C(6)-C(5)-C(4)	120.34(15)
C(6)-C(5)-H(5A)	119.8
C(4)-C(5)-H(5A)	119.8
C(5)-C(6)-C(1)	120.58(16)
C(5)-C(6)-H(6A)	119.7
C(1)-C(6)-H(6A)	119.7
O(1)-C(7)-O(2)	123.60(16)
O(1)-C(7)-C(1)	123.80(18)
O(2)-C(7)-C(1)	112.58(15)
N(1)-C(8)-C(4)	112.60(11)
N(1)-C(8)-C(9)	108.25(13)
C(4)-C(8)-C(9)	112.58(13)
N(1)-C(8)-H(8A)	107.7
C(4)-C(8)-H(8A)	107.7
C(9)-C(8)-H(8A)	107.7
C(10)-C(9)-C(8)	111.15(14)
C(10)-C(9)-H(9A)	109.4
C(8)-C(9)-H(9A)	109.4
C(10)-C(9)-H(9B)	109.4
C(8)-C(9)-H(9B)	109.4
H(9A)-C(9)-H(9B)	108.0

O(3)-C(10)-C(9)	105.97(16)
O(3)-C(10)-C(11)	113.01(16)
C(9)-C(10)-C(11)	109.70(15)
O(3)-C(10)-H(10A)	109.4
C(9)-C(10)-H(10A)	109.4
C(11)-C(10)-H(10A)	109.4
C(12)-C(11)-C(10)	111.64(15)
C(12)-C(11)-H(11A)	109.3
C(10)-C(11)-H(11A)	109.3
C(12)-C(11)-H(11B)	109.3
C(10)-C(11)-H(11B)	109.3
H(11A)-C(11)-H(11B)	108.0
C(11)-C(12)-N(1)	110.92(13)
C(11)-C(12)-H(12A)	109.5
N(1)-C(12)-H(12A)	109.5
C(11)-C(12)-H(12B)	109.5
N(1)-C(12)-H(12B)	109.5
H(12A)-C(12)-H(12B)	108.0
O(2)-C(13)-H(13A)	109.5
O(2)-C(13)-H(13B)	109.5
H(13A)-C(13)-H(13B)	109.5
O(2)-C(13)-H(13C)	109.5
H(13A)-C(13)-H(13C)	109.5
H(13B)-C(13)-H(13C)	109.5
O(3)-C(14)-C(15)	109.5(2)
O(3)-C(14)-H(14A)	109.8
C(15)-C(14)-H(14A)	109.8
O(3)-C(14)-H(14B)	109.8
C(15)-C(14)-H(14B)	109.8
H(14A)-C(14)-H(14B)	108.2
C(14)-C(15)-H(15A)	109.5
C(14)-C(15)-H(15B)	109.5
H(15A)-C(15)-H(15B)	109.5
C(14)-C(15)-H(15C)	109.5
H(15A)-C(15)-H(15C)	109.5
H(15B)-C(15)-H(15C)	109.5

14.Table S5. Torsion angles [deg] for 15.

C(6)-C(1)-C(2)-C(3)	1.3(3)
C(7)-C(1)-C(2)-C(3)	-176.54(16)
C(1)-C(2)-C(3)-C(4)	0.3(3)
C(2)-C(3)-C(4)-C(5)	-2.0(2)
C(2)-C(3)-C(4)-C(8)	173.69(15)
C(3)-C(4)-C(5)-C(6)	2.0(2)
C(8)-C(4)-C(5)-C(6)	-173.52(16)
C(4)-C(5)-C(6)-C(1)	-0.4(3)
C(2)-C(1)-C(6)-C(5)	-1.3(3)
C(7)-C(1)-C(6)-C(5)	176.48(16)
C(13)-O(2)-C(7)-O(1)	1.7(3)
C(13)-O(2)-C(7)-C(1)	-176.60(17)
C(6)-C(1)-C(7)-O(1)	175.29(18)
C(2)-C(1)-C(7)-O(1)	-6.9(3)

C(6)-C(1)-C(7)-O(2)	-6.5(2)
C(2)-C(1)-C(7)-O(2)	171.35(15)
C(12)-N(1)-C(8)-C(4)	-174.91(14)
C(12)-N(1)-C(8)-C(9)	59.98(17)
C(3)-C(4)-C(8)-N(1)	122.92(16)
C(5)-C(4)-C(8)-N(1)	-61.5(2)
C(3)-C(4)-C(8)-C(9)	-114.36(18)
C(5)-C(4)-C(8)-C(9)	61.19(19)
N(1)-C(8)-C(9)-C(10)	-59.66(19)
C(4)-C(8)-C(9)-C(10)	175.21(15)
C(14)-O(3)-C(10)-C(9)	-167.24(18)
C(14)-O(3)-C(10)-C(11)	72.6(2)
C(8)-C(9)-C(10)-O(3)	-65.54(18)
C(8)-C(9)-C(10)-C(11)	56.8(2)
O(3)-C(10)-C(11)-C(12)	63.90(19)
C(9)-C(10)-C(11)-C(12)	-54.1(2)
C(10)-C(11)-C(12)-N(1)	55.0(2)
C(8)-N(1)-C(12)-C(11)	-58.60(18)
C(10)-O(3)-C(14)-C(15)	166.4(2)

15.Table S6. Hydrogen bonds for 15 [A and deg.].

D-H...A	d(D-H)	d(H...A)	d(D...A)	<(DHA)
N(1)-H(1B)...Cl(1)	0.90	2.23	3.0807(14)	156.8
N(1)-H(1A)...Cl(1)#1	0.90	2.25	3.1402(14)	172.8

Symmetry transformations used to generate equivalent atom
